# Supplementary material for: Mapping Phenomena Relevant to Adolescent Emotion Regulation: A Text-Mining Systematic Review
Source: Adolesc Res Rev. 2021 May 21;7(1):127–39. doi: 10.1007/s40894-021-00160-7 (PMC8138847; doi:10.1007/s40894-021-00160-7)
Supplement: Supplementary file 1 — Supplementary file1 (ZIP 54379 kb) [file 40894_2021_160_MOESM1_ESM.zip › veni_sysrev-master/action_letter_final.html]

Action letter


# Action letter

#### Caspar J. van Lissa

#### 4/6/2021

**Comment 1:** *Please do be sure to “unblind” your manuscript, if needed.*

**Response:** I have done as requested.

**Comment 2:** *Title: Please capitalize the first letters of key words (look at a few articles in the journal or check with the APA manual). Ensure that it is the same every time you enter it into the editorial manager. It should appear the same ever time it appears in the pdf that you approve. Also, do not use bold. …. Well, given where we are with the manuscript, I might as well give you exactly what I need to have you upload: Mapping Correlates of Adolescent Emotion Dysregulation: A Text-mining Systematic Review*

**Comment 3:** *Page 1, remove this at the bottom: Word count: 6776*

**Response:** I have done as requested.

**Comment 4:** *THROUGHOUT: use “toward” rather than “towards”*

**Response:** I have done as requested.

**Comment 5:** *In the abstract and throughout: Remove the use of the first person (we, our). [I note this in case you use it in your revision.]*

**Response:** This has been done.

**Comment 6:** *THROUGHOUT: use youth instead of youths. We consider youth plural. Thanks. [I note this in case you use it in your revisions.]*

**Response:** I don’t believe I made this mistake. I did find one instance of “youth”.

**Comment 7:** *Abstract, line 24 of the pdf, change “papers” to this: studies Abstract, avoided stilted language. Begin sentence with The results rather than Results.*

**Response:** Respectfully, I do not understand the first comment. I have changed “Results” to “The results”. Please let me know if further action needs to be taken here.

**Comment 8:** *Abstract, line 43 of the pdf, replace “This” with something more specific, perhaps something like: The findings illustrate …*

**Response:** I have done as requested.

**Comment 9:** *Abstract: remove this: (TMSR)*

**Response:** I have done as requested.

**Comment 10:** *Page 2, at the top, remove the title and insert this level 1 heading: Introduction These are the typical headings that typesetters will be able to work with:*

**Response:** I have done as requested.

**Comment 11:** *Level 1: Centered, Bold, Upper and Lower Case Level 2: Flush Left, Bold, Upper and Lower Case Level 3: Flush left, bold, Initial cap/lower case, period. Run in text Level 4: Flush left, bold italic, Initial cap/lower case, period. Run in text Level 5 Paragraph indent, nobold italic, Initial cap/lower case, period. Run in text*

**Response:** I have done as requested.

**Comment 12:** *As a reminder, you need at least two subheadings if you are going to have one.*

**Response:** I have removed the offending subheading; it was not essential.

**Comment 13:** *Throughout, please avoid string (and even most double) citations. If your cite is important, you should have more text to go with it; no need for redundancy if it can be avoided. Also avoid dated citations unless it is a classic or central to your arguments. I know that this will be a challenge for you as you are trying to give readers a sense of the field. Please rest assured that I am ok with the overall number of citations (I have no issue with that), it is when you cite multiple citations for the same proposition. A way around this issue is to increase specificity in what you present, and then cite to the appropriate sources. Or, of course, simply deleting some citations also would work. [Generally, do avoid citations from the last century.] Please do see what you can do in this regard.*

**Response:** I have removed string citations throughout. In some cases, I have left only essential references; in other cases I have split up the string and explicated the specific contribution of each reference. The only remaining exception is a situation where I specifically refer to two systematic reviews, and must thus cite them both.

**Comment 14:** *Another consideration, depending on how you edit this draft: Have the author names in parentheses. Just state the proposition and have the cite at the end of it; we do not want to focus readers on the names of authors. [The exception to that could be when you refer to someone’s theory that needs to have the author’s name. Otherwise, authors should remain in the back ground.] Avoid name-dropping. Studies should be evaluated on their merit, not who did them.*

**Response:** I have done as requested throughout the manuscript.

**Comment 15:** *I need a different introductory paragraph. This is what I now ask of authors: I need an introductory paragraph that begins by noting the topic, its importance, the research gap, and a general statement about how you will address the research gap. That is, the first paragraph works best with a closing sentence that begins with something like: This study….. Then, in the paragraphs that follow, we’d expect to have you go straight to reviewing the relevant research. Try to keep the introductory paragraph to the introductory literature review to one full paragraph that really gets to the point. [One strong paragraph is preferred; do two only if you absolutely have to. …. Save specifics and nuances for the literature review. Here, you are just introducing the topic. Clear?]*

**Response:** I have done as requested. Note that I deviate from the recommended structure in one respect: As the previous Reviewer’s comments emphasized the importance of clarifying the rationale behind the methods used, I have retained the newly written paragraph explaining the approach after the opening paragraph. The structure is thus: 1) Topic 2) Importance 3) Research gap 4) How you will address the research gap 5) (New paragraph) Rationale behind the method This seems like an appropriate structure for a substantive paper that also introduces a methodological innovation - but please let me know if you feel differently.

**Comment 16:** *Also regarding the introductory paragraph, avoid tangents. As an example, the first sentence of the first paragraph is not needed. In fact, much of the first paragraph could be trimmed to one sentence. Several of the other introductory paragraphs also can go, or be integrated later. Again, you are just introducing here.*

**Response:** In rewriting the introductory paragraph as requested in the previous comment, I have made sure to avoid tangents.

**Comment 17:** *Page 4, use a more specific heading than “Existing theoretical landscape”. […] So, given that, the heading should be something like: Theoretical Approaches to Emotional Regulation. It should be a level 2 heading.*

**Response:** I have done as requested.

**Comment 18:** *Also, in the section below it, you have too much tangential information. Just present the relevant theories that are influencing research at this point in time. Just describe the theories as they are, focus on those that relate directly to emotional regulation. We don’t need history or to know much about where they come from. Just describe the theories as they relate to the issue at hand.*

**Response:** I have tried to make the requested cuts throughout the theory section. Note that the previous Reviewer had asked me to provide more of an “authoritative” narrative in this section. To do so, I have contextualized the micro-level theories within the macro frameworks like Bronfenbrenner’s and Sameroff’s models. I assumed that this is not what you mean by “tangential information”, and left these additions intact.

**Comment 19:** *There should not be a need for the first subheading on page 8 (plus, you would have needed two at the same level). The theory section should end up shorter.*

**Response:** I have removed this subheader, as requested.

**Comment 20:** *Page 8, the new section beginning on line 48: the topic sentence of the paragraph should be about the paragraph; remove the first part that focuses on the part before.*

**Response:** I have done as requested.

**Comment 21:** *Right before that section, you have something about “The present study lays…”. I need you to remove sentences like that. What the present study does should be presented in the current study section, it should not be interspersed in the literature review introducing the study. The literature review does just that, review. It should lead to the research questions/hypotheses; it is just laying a foundation.*

**Response:** I have moved this sentence to “The Current Study” section as requested.

**Comment 22:** *Page 10, center the heading, and have it read like this: The Current Study*

**Response:** I have done as requested.

**Comment 23:** *I need an edit to this section. The current study section should briefly note the research gap, note aims/expectations/hypotheses/research questions and briefly remind readers of the rationales for them. The rationales should be obvious from the literature review above it (you should not introduce new research/cites in this section).*

**Response:** I have rewritten “The Current Study” according to these specifications.

**Comment 24:** *Ironically, the first sentence or so of the Methods section is what should be in the current study section.*

**Response:** This sentence has been moved to “The Current Study”.

**Comment 25:** *Page 12, line 46, this should not be a level 1 heading.*

**Response:** I have changed it to level 2.

**Comment 26:** *These are the level 1 headings that we need to have: Abstract Introduction Current Study Methods Results Discussion Conclusion Acknowledgments*

**Response:** All these headers are now present.

**Comment 27:** *Page 17, I need a different introductory paragraph to the discussion.* The first paragraph of the discussion should start more broadly and give readers a good sense of the issues that need to be addressed in this area, as well as highlight the complexities/questions involved and GENERALLY how you addressed them (such as in one sentence at the end of the paragraph). Assume, for example, that readers have skipped much of your study and jumped straight to the discussion section. In the sentences after that, present what you found and the importance of your contribution (and limitations and so forth). I apologize for this journal style edit.

**Response:** I have rewritten the introductory paragraph and remainder of the Discussion as requested.

**Comment 28:** *Page 23, line 29, you should not have a paragraph that begins with “To conclude….”and then follow that with two more paragraphs. Conclusions should conclude, should be the last word.*

**Response:** Thank you, good point. I have removed this in rewriting the Discussion and Conclusion.

**Comment 29:** *Conclusion: do not start with “In sum,” It should stand on its own. Much of what you have in the conclusion is implications. I need it more conclusory.*

**Response:** I have removed this turn of phrase and rewritten the Discussion and Conclusion. Implications are now in a separate subsection, and the Conclusion is more conclusory.

Comments: Your current conclusion has much in it that should be in the discussion. Here is what I typically tell authors regarding what I need in the conclusion. Insert this level 1 heading before it: Conclusion Begin by (briefly) reminding the reader of the topic, the research gap, and how you addressed it. Then go to what you found and its importance. Be sure to state your findings very clearly. The implications can focus on many aspects of the study, such as the practice implications (briefly). But, for us, the implications also should include a focus on the understanding of adolescence/early adulthood/emerging adulthood and the contribution to that research. The bulk of the paragraph, though, should be focused on the key findings. Do try to have it be just one strong paragraph.

**Response:** I have restructured the Conclusion according to these specifications. The previous conclusion has been merged with the Discussion; there was much redundancy. Thank you for spotting this.

**Comment 30:** *Page 23, line 50 of the pdf: delete the sentence.*

**Response:** I have done as requested.

**Comment 31:** *You have figures with color. I would like to note at this point that when we get to proof stage, and I sure hope we get to that stage, you will be asked to pay more for the use of color. Please be sure to read that very carefully. It is NOT asking you to pay more for what will be electronic copies; it asks you to pay more for the actual print copies. Just decline to pay for the print copies. I note this because authors often misread and then try to edit their text so they do not need to pay a hefty sum of money. There is no need for that. Just read it carefully. Of course, if you wish to pay, then that is up to you.*

**Response:** Understood, will do. I’ve also adapted the plots so they will still be intelligible when printed in grayscale, or when viewed by the color-blind.

**Comment 32:** *You acknowledgment page (the one to be uploaded separately) needs to report more information.*

**Response:** I have included all requested information.
