# Supplementary material for: Mapping Phenomena Relevant to Adolescent Emotion Regulation: A Text-Mining Systematic Review
Source: Adolesc Res Rev. 2021 May 21;7(1):127–39. doi: 10.1007/s40894-021-00160-7 (PMC8138847; doi:10.1007/s40894-021-00160-7)
Supplement: Supplementary file 1 — Supplementary file1 (ZIP 54379 kb) [file 40894_2021_160_MOESM1_ESM.zip › veni_sysrev-master/~WRL0005.tmp]

Mapping Phenomena Relevant to Adolescent Emotion Regulation: A Text-mining Systematic Review

Caspar J. van Lissa^1,2^

^1^ Utrecht University faculty of Social and Behavioral Sciences, department of Methodology & Statistics

^2^ Open Science Community Utrecht

Author note

Correspondence concerning this article should be addressed to Caspar J. van Lissa, Padualaan 14, 3584CH Utrecht, The Netherlands. E-mail: [c.j.vanlissa@uu.nl](mailto:c.j.vanlissa@uu.nl)

Abstract

Adolesence is a developmentally sensititve period for emotion regulation with potentially lifelong implications for mental health and well-being. Although substantial empirical research has addressed this topic, the literature is fragmented across subdisciplines, and an overarching theoretical framework is lacking. The first step toward constructing a unifying framework is identifying relevant phenomena. This systematic review of 6305 articles used text mining to identify phenomena relevant to adolescents’ emotion regulation. First, a baseline was established of relevant phenomena discussed in theory and recent narrative reviews. Then, article keywords and abstracts were analyzed using text mining, examining term frequency as an indicator of relevance and term co-occurrence as an indicator of association. The results reflected themes commonly featured in theory and narrative reviews, such as socialization and neurocognitive development, but also identified undertheorized themes, such as developmental disorders, physical health, external stressors, structural disadvantage, substance use, identity and moral development, and sexual development. The findings illustrate how text mining systematic reviews, a novel approach, may complement narrative reviews. Future theoretical work might integrate these undertheorized themes into an overarching framework, and empirical research might consider them as promising areas for future research, or as potential confounders in research on adolescents’ emotion regulation.

*Keywords:* emotion regulation, adolescence, systematic review, text mining, machine learning

**Introduction**

Adolescence is a developmentally sensitive period for emotion regulation (Zimmermann & Iwanski, 2014). Although most adolescents successfully develop mature emotion regulation abilities, as many as one in five develop psychopathology (Lee et al., 2014) with roots in emotion regulation difficulties (Aldao, Nolen-Hoeksema, & Schweizer, 2010). Given the prevalence of emotion regulation difficulties in adolescence, their implications for mental health and downstream cost to society, it is important to have a comprehensive overview of the phenomena associated with adolescents’ emotion regulation. Despite the abundance of publications in this field, it is difficult to obtain a comprehensive understanding of emotion regulation in adolescence (Riediger & Klipker, 2014): Different (sub)disciplines have approached the topic in disparate ways (Riediger & Klipker, 2014), without consistent terminology (Bariola, Gullone, & Hughes, 2011), conceptual frameworks (Stifter & Augustine, 2019), or an overarching theoretical framework (Buss, Cole, & Zhou, 2019). This has prompted calls for the integration of knowledge across research areas (Riediger & Klipker, 2014) and consolidation of empirical research into overarching theory (e.g., Buss et al., 2019). Formal methods for theory construction state that the first step in this endeavor is to identify well-established relevant *empirical phenomena*, defined as stable and general features of the world (Borsboom, Dalege, Kievit, & Haig, 2020). The current study used a text mining systematic review to provide a comprehensive map of the empirical phenomena relevant to adolescent emotion regulation.

Currently, few instruments exist for the identification of relevant phenomena; instead, theorists typically rely on expert opinion (Borsboom et al., 2020). This article argues that systematic reviews are a suitable and relatively more objective method for phenomena detection. Traditional narrative reviews are limited, however, by small convenience samples, confirmation bias, and an undue emphasis on positive results (Littell, 2008). These limitations are overcome by a novel method: the text mining systematic review (TMSR). Relative to narrative reviews, text mining can cover arbitrarily greater corpora, and gleans insights from the literature by means of a relatively more transparent, objective, and reproducible process. This approach assumes the frequency with which phenomena recur in the literature to be indicative of their relevance. If it is additionally assumed that the frequency with which phenomena are studied together is indicative of the extent to which they are thought to be associated, then the co-occurrence of phenomena within publications can be interpreted as a rudimentary nomological network: a mapping of relationships between theoretically relevant phenomena (see Alavi, Archibald, McMaster, Lopez, & Cleary, 2018). Such a network can serve as a starting point for the formulation of a proto-theory, which would additionally involve abductive reasoning: specifying putative mechanisms to explain associations between the phenomena (Borsboom et al., 2020). Text mining systematic reviews constitute an inductive and exploratory approach: Their aim is phenomena detection based on generalization from observations (data) without appeal to theory (Haig, 2005). By identifying and mapping the phenomena relevant to adolescent emotion regulation, such an inductive approach lays the groundwork for future theory development.

## Theoretical Approaches to Emotion Regulation

Although it has been argued that there is a paucity of theories *specific to* adolescent emotion regulation (see Buss et al., 2019), several *relevant* theories are commonly invoked in empirical work. A brief review of the existing theoretical landscape is provided here to contextualize the present systematic review and assess to what extent phenomena identified through text mining mirror or complement existing theory. Others have published detailed reviews of theories of emotional development (Buss et al., 2019) and of empirical research on adolescent emotion regulation (Riediger & Klipker, 2014). The left panel of Figure 1 visually summarizes the phenomena discussed in the theoretical literature reviewed below, along with whether these were discussed as putative causes, outcomes, indicators, or protective factors in relation to emotion regulation. This establishes a baseline for the original contributions of the text minning analysis.

Two of the most general theories invoked to frame developmental research are the bioecological model (Bronfenbrenner & Morris, 2007), and the transactional model (Sameroff, 2010). The bioecological model describes how the environment shapes individual development. At the individual level, every person is imbued with biological predispositions, and develops over time in interaction with contextual influences. The most immediate source of contextual influences is the microsystem, composed of primary caregivers and other close ties. Other environmental influences originate in the macrosystem - including political and economic influences - and the exosystem, consisting of cultural norms and values. The transactional model (Sameroff, 2010) is compatible with the bioecological model, but places a stronger emphasis on development as a product of reciprocal influences between child and environment. The transactional model distinguishes between proximal influences, which roughly correspond to the microsystem in the bioecological model, and distal influences, which derive from structural and environmental factors indirectly shaping development, like socio-economic status, schools, and the community (macro- and exosystem). With increasing age, these distal influences gain ground on proximal influences. These two theories have a broader scope than most. This means that they can be invoked to contextualize any developmental study, but lack specificity - a shortcoming that curtails a theory’s utility in generating hypotheses. Domain-specific theories, by comparison, offer greater specificity.

One such domain-specific theory is Hall’s notion of “storm and stress” in adolescence (see Arnett, 1999). It describes how hormonal changes diminish self-control and increase reactivity, which in turn leads to emotion regulation difficulties, increased conflict with parents, and risky behavior. Contemporary theories recast this notion of diminished self-control and increased emotional reactivity as a normative change that facilitates emotional maturation at the risk of emotional disturbance (Arnett, 1999). A potential limitation of conceptualizing emotion regulation difficulties as normative is that this underemphasizes heterogeneity in developmental trajectories (cf. Crone & Dahl, 2012).

One theory of normative emotional development focuses on developmental influences to a greater extent (Sroufe, 1995). It posits that, as children grow older, their increasing self-regulatory abilities drive a transition from external emotion regulation by primary caregivers toward autonomous emotion regulation. This theory emphasizes social and cognitive influences as drivers of emotion regulation development. Social influences mainly occur through parental co-regulation, parenting behaviors, and parent-child attachment. Cognitive influences occur through the development of the central nervous system (CNS), cognition, and self-regulation. This theory’s relevance for adolescent research is somewhat diminished by its focus on early childhood. However, research indicates that there is some continuity in developmental influences throughout childhood and adolescence; for instance, mothers’ and fathers’ unique roles in emotional development are similar in childhood and adolescence (see Van Lissa & Keizer, 2020; Van Lissa, Keizer, Van Lier, Meeus, & Branje, 2019). The emphasis on socialization and neurocognitive development, which is evident from both theories, is the focus of several theories with a narrower scope.

One widely cited theory of socialization is the tripartite model (Morris, Silk, Steinberg, Myers, & Robinson, 2007). It describes three pathways through which parents shape emotion regulation: modeling, parenting practices, and the emotional family climate, which subsumes attachment and marital relationship quality. This theory acknowledges unique contributions by mothers and fathers, and the importance of siblings. Others have adapted the tripartite model to describe peer socialization (Reindl, Gniewosz, & Reinders, 2016), thus illustrating its wider usefulness. A more abstract take on socialization is found in the internalization model of emotional development (Holodynski & Friedlmeier, 2006). This theory describes the socialization of the role of emotion in communication, and the cultural symbolic function of emotion.

In research on neurocognitive aspects of adolescents’ emotion regulation, polyvagal theory has been influential (Porges, 1995). This theory examines emotional experience and -regulation in relation to autonomous nervous system functioning, respiratory sinus arrhythmia, and the stress response. Although polyvagal theory is relevant for development, it does not explicitly address it. Among developmental neurocognitive theories, there is some consensus that the developmental asymmetry between motivational-emotional and inhibitory brain circuits gives rise to a “maturity gap” in middle adolescence (Crone & Dahl, 2012). According to the model of social-affective engagement and goal flexibility (Crone & Dahl, 2012), adolescents’ cognitive engagement is dynamically responsive to social and motivational goal salience. This flexibility prepares adolescents to develop mature regulatory abilities, but also places them at risk of greater impulsivity - for example, in pursuit of peer approval. To a greater extent than related writings (cf. Cracco, Goossens, & Braet, 2017), this model focuses on adolescents diverging destinies; why some youngsters flourish while others languish (Crone & Dahl, 2012). This is important for understanding individual differences in development. Although the relevance of this theory is enhanced by its focus on adolescence, it only tangentially addresses emotion regulation. Furthermore, whereas this theory addresses cognitive factors and peers as drivers of development, it underemphasizes other relevant phenomena, such as parenting (cf. Morris et al., 2007). Micro-scale theories like these are useful in explaining specific phenomena in detail, but do not provide a comprehensive understanding of adolescent’s emotion regulation. To this end, it is important to consider them in the context of a larger framework of relevant phenomena.

Theories of emotion regulation offer further insight into intra-individual drivers of emotion regulation development. The influential process model describes emotion regulation throughout the progression from eliciting cue to ultimate response (Gross, 2013). This model posits that individuals use strategies to modulate this process at different stages, consciously or otherwise. Maladaptive emotion regulation strategies tend to result in more negative emotions, diminished well-being, and greater strain in interpersonal relationships (Bell & Calkins, 2000). However, comparative studies indicate that the adaptive versus maladaptive psychosocial consequences of specific strategies appear to be partly contingent on the cultural context (see Bariola et al., 2011). Similar to the process model, the “social information processing” theory also describes the role of cognitive processes and strategies in emotion regulation (Lemerise & Arsenio, 2000). One shortcoming of these theories for understanding adolescent emotion regulation is the lack of a developmental component.

Despite the abundance of theory relevant to emotion regulation in adolescence, the literature has several limitations. First, few theories have explicitly addressed adolescence. As this life stage differs qualitatively from both childhood and adulthood, it is questionable whether theories focused on different age groups can be generalized to adolescents (Bariola et al., 2011). Furthermore, few theories have comprehensively addressed important predictors of development in this life stage, and none directly guide contemporary research in the field (Buss et al., 2019). Finally, existing theories vary widely in scope: Some are broad and non-specific; others describe a specific phenomenon in detail, but lack a broader perspective. Broad theories can be used to frame a wide range of research, and specific theories are more suitable for deductive hypothesis generation. To combine the strength of both, it would be beneficial to bridge these levels of analysis. There is a need for integrative theory formation, which could provide a unified framework to guide future empirical work.

## Prior Narrative Reviews

Literature reviews can shed light on undertheorized relevant phenomena by synthesizing inductive insights emerging from the empirical literature. This is especially important given the noted absence of overarching theory (see Buss et al., 2019). One recent narrative review, in exploring which factors contribute to adolescents’ emotion regulation development, clearly reflected the emphasis on neurocognitive factors and socialization that is evident from the theoretical literature (Riediger & Klipker, 2014). Other recent narrative reviews complement the theoretical literature to a greater extent by addressing relevant but undertheorized phenomena (Bariola et al., 2011; Coe-Odess, Narr, & Allen, 2019). The right panel of Figure 1 illustrates in what respects these narrative reviews reflect existing theory, and in what respects they complement it.

One seminal review discussed relevant phenomena at different levels of analysis (Bariola et al., 2011). At the individual level, such relevant phenomena include temperament and biological factors, like neurocognitive development and genes. At the level of proximal influences, the authors discuss socialization and modeling by parents, teachers, and peers. Finally, at the level of distal influences, culture and the media are discussed. This review also called for research on emotion regulation beyond early childhood and into adolescence, and on fathers’ unique contributions - a topic receiving increasing interest (see Pleck, 2004). In line with these recommendations, several recent publications have investigated mothers’ and fathers’ unique roles in emotion regulation socialization from childhood to adolescence (see Van Lissa & Keizer, 2020) and throughout adolescence (see Van Lissa et al., 2019).

As the aforementioned review is now a decade old, it is worth considering a more recent review for additional insight (Coe-Odess et al., 2019). This review offers a nuanced discussion of several additional topics. At the individual level, this includes the implications of physiological changes, including neurocognitive development and pubertal maturation. Pubertal development also precipitates sexual and romantic behavior, and the intensification of both biological sex differences and gender stereotyped behavior. This, in turn, likely modulates proximal influences through peers. The review further describes how individual hormonal changes intensify the stress response, which relates to adolescents’ greater susceptibility to emotion regulation difficulties. Finally, going beyond the implications of cognitive development discussed in other publications, the authors discuss how cognitive development and increased capacity for abstract thought relate to identity formation and increased emotional understanding. These themes relate to two key challenges in adolescence, namely identity (Meeus, 2011) and empathy development (Van Lissa et al., 2014). At the level of proximal influences, the review expands on the role of conflict with parents, which peaks in adolescence as youth become increasingly individuated. This is relevant because parent-adolescent conflict has been shown to impact both day-to-day mood swings and dispositional emotion regulation (see Van Lissa, Hawk, Koot, Branje, & Meeus, 2017). The review further describes how adolescents become increasingly oriented toward peers, which increases their sensitivity to social status and norms, and prompts concomitant increases in peer pressure and risk taking. The review devotes limited attention to distal influences.

There are notable parallels between phenomena relevant to adolescents’ emotion regulation as identified in these narrative reviews, and in the preceding theoretical literature, as can be seen in Figure 1. Nevertheless, these literature reviews also touch upon issues that have received little attention in theoretical work. This illustrates the general principle that reviews of the empirical literature can contribute inductive insight into relevant phenomena underrepresented in theory. An important shortcoming is that all reviews in this field have been unstructured narrative reviews; a method that is often limited in scope and susceptible to bias (Littell, 2008).

# The Current Study

The empirical literature on adolescent emotion regulation is somewhat fragmented accross subdisciplines, and lacks an overarching theoretical framework. The current study seeks to integrate this body of work by means of a comprehensive text mining systematic review (TMSR). This is an inductive study, and as such, does not test hypotheses. The aims of the study are to identify the most relevant phenomena in the literature on adolescent emotion regulation, based on the frequency with which phenomena are reported, and to map potential relationships between phenomena, based on their co-occurrence within studies. The resulting map of relevant phenomena helps integrate the empirical literature. Moreover, as identifying relevant empirical phenomena is the first step in theory construction methodology, this study lays the groundwork for future theory development.

# Methods

This text mining systematic review considered the frequency with which a phenomenon is covered in the literature, or *term frequency*, to be indicative of its relevance. Term frequency can be rank ordered to identify the most relevant phenomena. Furthermore, the frequency with which phenomena are investigated together within publications, or *term co-occurrence*, is considered to be indicative of a putative relationship between them. These two metrics can be jointly visualized as a network graph, thereby “mapping” phenomena relevant to adolescent emotion regulation.

Inductive research affords the researcher with substantial creativity (Wagenmakers et al., 2018). This means that subjective decisions are made throughout the analysis process. To ensure that all such decisions are properly documented, all code, data, and the historical record of this project are available in a public research repository at <https://github.com/cjvanlissa/veni_sysrev>. The Workflow for Open Reproducible Code in Science was used to make all analyses reproducible (WORCS, Van Lissa et al., 2020). Reuse of the analysis code and secondary analysis of the data are encouraged.

## Search Strategy

The search was conducted in Web of Science, the most comprehensive database accessible to the lead author with permissions to export keywords and abstracts. The search strategy was based on procedures described by Staaks (Staaks, 2019). First, a reference set of 29 articles was compiled. Then, a search string was constructed to retrieve the articles in this set. The search string consisted of synonyms of emotion regulation and adolescence. It returned 6653 results, including 25 records in the reference set. To match all 29 reference set items required adding the terms "emotio* socialization" OR "emotio* processes" as synonyms for emotion regulation. Doing so resulted in 191 more hits, most of which did not meet the inclusion criteria explained below. These terms thus appeared to be overly inclusive, and the original search string was used.

## Screening

Starting with all 6653 records identified through Web of Science, duplicates were removed based on DOI matches (n = 2) and title similarity (n = 54). Rayyan QRCI (Ouzzani, Hammady, Fedorowicz, & Elmagarmid, 2016) identified an additional 13 duplicates. Articles were screened based on two main criteria: They had to address emotion regulation or a synonymous construct, and the target population must overlap with the age range of adolescence (10-24, as defined by Steinberg, 2014). Preliminary screening was conducted in Rayyan. After 559 articles were screened (192 excluded), screening continued in the free open source program ASReview (Van de Schoot et al., 2020). This program uses machine learning to screen articles; the algorithm used in the present study was “naive Bayes”. An additional 541 articles were screened (85 excluded), until among the most recently screened 100 articles only 6 were excluded. In total, 6305 articles were deemed suitable for analysis.

## Analysis 1: Author Keywords

The corpus for this first analysis consisted of author-provided keywords. Keywords were extracted by document, and an exclusion filter of methodological terms and similar non-substantive words was applied. The resulting corpus consisted of 5031 documents with 8080 unique terms. One important step in reviewing the literature is to examine heterogeneity of the corpus; to determine whether there is, for example, a clear divide between psychiatric and developmental texts. To this end, topic modeling using latent dirichlet allocation was conducted (Blei, Ng, & Jordan, 2003). However, as no subcorpora could be identified (see [online supplement](https://github.com/cjvanlissa/veni_sysrev/blob/master/topic_models.md)), the sample was analyzed in its entirety.

**Identifying common terms.** To identify what phenomena are represented in this corpus, terms occurring in the largest number of texts were analyzed. To classify closely related terms, a dictionary was used that described 108 terms using 464 regular expression queries. This dictionary and the classification function are available for reuse. After dictionary encoding, the remaining number of unique terms was 5292. To reduce the number of terms to a more manageable set, word frequency was modeled with a negative binomial distribution. Terms exceeding the ${97.5}^{th}$ percentile were retained. This corresponded to terms occurring in at least 21 documents. This is a subjective criterion, but compared to the common practices of either retaining a fixed number of terms or pruning terms below a fixed frequency (see Benoit et al., 2018), it has the advantage of being responsive to the empirical distribution of term frequencies. Note that the vast majority of pruned terms (4004) occurred only once in the corpus. Pruning resulted in 84 remaining terms, which occurred in 4827 documents. The issues covered in this body of literature are visualized in Figure 2.

**Mapping the literature.** To map the literature, a term co-occurrence matrix was computed, which represents how frequently words occurred within the same document (see Figure 3). In total, there were 2498 co-occurrence relationships. To aid interpretability, small coefficients were again pruned using a negative binomial distribution, retaining co-occurrences exceeding the ${97.5}^{th}$ percentile. Note that this is a subjective criterion, which corresponded to terms that co-occurred in more than 25 documents. After pruning, 106 co-occurrence relationships remained.

Following the procedure described in the introduction to classify phenomena discussed in the theoretical literature, each of the remaining terms were categorized as either a putative cause, outcome, protective factor, or indicator of emotion regulation. Note that this classification is based on a subjective reading of the literature, and that the nature of associations between phenomena is likely to be more complex than presented here. For example, some of these associations are likely to be spurious, or bidirectional (e.g., emotion regulation is known to both predict and be predicted by conflict with parents, Van Lissa et al., 2017). This classification is merely intended as a starting point for further reflection on the potential role of each construct.

## Analysis 2: Abstract text mining

The corpus for this second analysis consisted of the abstracts of the selected articles. Keywords, as examined in Analysis 1, convey high-quality information because they are carefully chosen by authors to capture the essence of a study. However, as authors are typically limited to 5 keywords, some nuance may be lost. Abstracts, by contrast, offer greater freedom of expression, but present a greater challenge when it comes to extracting relevant information. It has been shown that retaining nouns and adjectives generally helps derive more interpretable text mining models (Martin & Johnson, 2015). For example, nouns help capture terms like “emotion”, and adjectives help capture the “mental” in “mental health”. The natural language processing technique “part-of-speech tagging” (POS-tagging) was used to identify each word’s grammatical function within the sentence context. Finally, stemming was used to reduce the retained terms to their root form; a common procedure to ensure that terms are correctly classified regardless of their use in a sentence. As in Analysis 1, heterogeneity was explored using latent dirichlet allocation - but no subcorpora were identified ([see online supplement](https://github.com/cjvanlissa/veni_sysrev/blob/master/topic_models.md)).

**Feature engineering.** When conducting text mining analysis on unstructured text data (as opposed to author key words), focusing on individual words out of context can reduce interpretability. For instance, the central construct of this review, “emotion regulation”, is already a bigram. To obtain more meaningful units of analysis, the textrank algorithm (Wijffels, 2019) was used to identify $n$-grams (with $n\leq3$). This is sufficient to capture trigrams, like “parent-child conflict”, but would not capture quadragrams like “parent-child conflict resolution”. The resulting $n$-grams were merged with the original data.

**Identifying common terms.** After applying the dictionary and exclusion filter as explained in Analysis 1, the resulting corpus consisted of 5097 documents with 11448 unique words. Again, the ${97.5}^{th}$ percentile of a negative binomial distribution was used as a subjective threshold for pruning the least common terms, which corresponded to terms occurring in more than 6 documents. The identified important keywords are displayed in Figure 2.

**Mapping the literature.** A term co-occurrence matrix was constructed following the procedure described in Analysis 1. In total, there were 850 co-occurrence relationships. Small coefficients below the ${97.5}^{th}$ percentile of a negative binomial distribution were again pruned. This is a subjective criterion, which corresponded to terms that co-occurred in more than 10 documents. After pruning, 43 co-occurrence relationships remained. Figure 3 displays the resulting co-occurrence matrix as a force directed graph.

# Results

In the analysis of author keywords (Analysis 1), emotion regulation and associated mental health-related outcomes were foremost among the common terms in the corpus (Figure 2). Other frequent terms reflect important themes discussed in the theoretical review of the literature; for instance, the terms *neural*, *parenting*, and *stress* correspond to themes discussed by Coe-Odess et al. (2019): Neurocognitive development, the role of the parents, and adolescents’ increased stress response. Importantly, the most common terms also include several concepts not featured prominently in the theoretical review. For example, *ADHD/CD* (cf. Braet et al., 2014), *substance* use (cf. Pierrehumbert et al., 2002), and *minority status* (cf. Myers, 2009) are common in the corpus, but featured less prominently in the theoretical review.

In the analysis of abstracts (Analysis 2), emotion regulation and associated mental health-related outcomes were evidently the most common terms. There was substantial agreement between the most common terms identified in the analysis of author keywords and abstracts. Specifically, $75\%$ of the most frequent terms identified in the author keywords were also present in the abstracts, and conversely, $89\%$ of the most frequent terms from the abstracts were present in the keywords. There were also some differences; for instance, the term *sex* was more frequent than in Analysis 1, suggesting that sex differences are regularly reported in Abstracts even if they are not mentioned in the keywords. The term *parenting*, which ranked highly in the keyword analysis, was displaced by *mothers* in the analysis of abstracts. This reflects the prior observation that parenting is most often operationalized in terms of mothering (Pleck, 2004).

With regard to the term co-occurrence analyses, the analysis of keywords (Analysis 1, see Figure 3) indicated that emotion regulation is evidently a central construct to which most other constructs were directly linked. This suggests that the search successfully identified factors relevant for adolescents’ emotion regulation. The remaining graph was notably sparse, with few interconnections between terms.

Compared to the keyword co-occurrence graph, the analysis of abstracts (Analysis 2) revealed an even sparser network. The structure also differed, as emotion regulation and mental health-related terms appeared to form a central axis. Many relevant terms were connected to this axis, but not directly to emotion regulation (as was the case in Analysis 1). The only terms directly connected to emotion regulation were *neural*, *mothers*, *attachment* and *ptsd*.

# Discussion

Although a substantial body of research has investigated emotion regulation in adolescence, this literature is somewhat fragmented across subdisciplines and lacks a unifying theoretical framework. The first step toward constructing such a unifying framework is identifying relevant empirical phenomena in this field. The present study set out to identify such relevant phenomena and map associations among them using a novel method: the text mining systematic review (TMSR).

The results of both analyses reflected some of the phenomena broadly considered to be relevant in prior theoretical literature and empirical reviews - particularly phenomena related to neurodevelopment and socialization. Other frequently occurring phenomena were mental health-related outcomes that involve emotion regulation difficulties (see Aldao et al., 2010). This finding is consistent with emotion regulation’s implication in a range of mental health problems (Lee et al., 2014), and underlines the importance of research in this area.

The analyses also identified several novel themes of relevant phenomena that are underrepresented in the theoretical literature and prior systematic reviews. One such theme pertains to developmental disorders, such as ADHD/CD and autism. This theme recurred in both analyses, although its constituent terms were ranked more highly in the analysis of keywords as compared to abstracts. Another theme revolved around adolescents’ physical health (sic), which was also reflected in terms like sleep, sports, and disability status. This finding indicates that there is substantial empirical work on emotion regulation and physical health, although physical health is underrepresented in theory. As with the theme of developmental disorders, terms related to physical health ranked more highly in the keyword analysis than in the abstract analysis. This might indicate that a broader vocabulary was used to describe physical health in the abstracts; a diverse vocabulary increases the difficulty of correctly classifying terms belonging to a theme. External stressors were another important theme, reflected in terms like bullying, stress, PTSD, abuse, violence, life events, historic events (e.g., earthquakes, war), parenting stress, and divorce. Based on the review of the theoretical literature, this indeed appears to be an under-theorized area. Conceptually, external stressors most closely align with the notion of the exosystem in the bioecological model (Bronfenbrenner & Morris, 2007), but they have rarely been discussed as such in the theoretical literature. The impression that external stressors are an undertheorized theme is reinforced by the fact that studies linking adverse life events to adolescent emotion regulation tend to reference mostly prior empirical work, but not a theoretical framework (e.g., Stikkelbroek, Bodden, Kleinjan, Reijnders, & Baar, 2016). Finally, one emergent theme that appears to be underrepresented in existing theory is structural disadvantage. This theme was reflected in terms like minority status and discrimination, disability status, socio-economic status, adoption status, and sexual diversity. The aforementioned developmental disorders are also relevant in this context, as neuroatypical individuals tend to experience social exclusion (Cappadocia, Weiss, & Pepler, 2012). Future theoretical work might address these shortcomings.

The analysis of abstracts identified three additional themes not represented in the analysis of keywords. The first of these themes revolved around addictive behavior (e.g., substance use and device use). The second theme pertained to identity and moral development, two central topics in adolescent research (Meeus, 2011) with a common root in theory (Lapsley, 2015). This theme is also reflected by the terms values and personality. A final emerging theme related to sexual development, as indicated by the terms (biological) sex, puberty, sexual diversity, and romantic relationships. Taken together, these insights illustrate how inductive reviews can identify themes that are underemphasized in existing theory and narrative reviews.

The co-occurrence analyses revealed that most phenomena were directly linked with emotion regulation in the keyword analysis, and with a central axis of emotion regulation and its mental health-related outcomes in the abstract analysis. The emergence of a central axis of emotion regulation and mental health-related outcomes again suggest that these phenomena are consistently studied together. This makes sense, given the central role of emotion regulation in the etiology of mental health problems (Lee et al., 2014).

Compared to the keyword analysis, the abstract analysis yielded a sparser network, with fewer selected terms. This is likely an artifact of the unstructured nature of abstracts, which introduces greater noise in the analysis. Thus, fewer terms will exceed the detection threshold. In line with this interpretation, exploratory analyses indicated that 99% of terms occurred only once in the abstract analysis, compared to only 78% in the keyword analysis. Despite the sparser network, there was substantial correspondence between the terms retained in both networks. This supports the validity of the findings, and suggests that automatic keyword extraction from abstracts can identify relevant constructs, and may be a suitable alternative to author-provided keywords.

Both co-occurrence networks revealed relatively few interconnections between phenomena, and several frequently occurring terms were pruned due to a lack of connectivity. This provides empirical supports for the prior anecdotal claim that this literature is somewhat fragmented (Riediger & Klipker, 2014). The absence of terms from the co-occurrence networks does not mean that these phenomena are unimportant, but rather, that they are not well-integrated in the broader literature on adolescents’ emotion regulation. Several of these unembedded phenomena represent emerging themes in the literature; as is the case for research on fathers (see Pleck, 2004), identity (Campbell, Zimmer-Gembeck, & Duffy, 2019), friendship and social support (Y. Wang et al., 2020), autonomy (Vrolijk, Van Lissa, Branje, Meeus, & Keizer, 2020), sexual risk (Brown et al., 2006), and loneliness (Spithoven, Bijttebier, & Goossens, 2017).

## Implications

This results of this text mining systematic review reflected several familiar phenomena that are well-represented in existing theory. This has implications for the validity of the method, as it suggests that text mining can indeed be used to map relevant themes in the literature. The results also revealed relevant phenomena that are under-emphasized in narrative reviews and theory, such as developmental disorders, physical health, external stressors, structural disadvantage, substance use, and identity-, moral-, and sexual development. This has implication for future empirical research: In designing a study, researchers typically rely on theoretical foundations - and under-theorized phenomena are at risk of being overlooked. The present study offers guidance regarding additional relevant phenomena to consider as potential confounders or contributing causes.

The results further revealed that several relevant phenomena were relatively unembedded: These scored highly on term frequency, but were sparsely connected to other terms in the co-occurrence networks, or pruned from the graph altogether. This finding has implications for future research, as unembedded terms indicate potential knowledge gaps. Future research might study these phenomena in conjunction with other more well-established constructs in order to bridge gaps in existing knowledge and bring these phenomena into the mainstream literature.

Additional implications arise from the structure of the co-occurrence graphs. Both analyses revealed close ties between emotion regulation and mental health-related outcomes, which is consistent with the role of emotion regulation in the etiology of mental health problems (see Lee et al., 2014). This has implications for the societal relevance of research in this area. Furthermore, both analyses revealed that most constructs were directly tied to emotion regulation and mental health, with few connections among constructs. These sparse networks validate prior anecdotal claims that the literature in this field is somewhat fragmented (Riediger & Klipker, 2014) and lacks an overarching theoretical framework (Buss et al., 2019). An important implication of this finding is that, indeed, work remains to be done to integrate this diffuse field, and to generate an overarching theory of adolescent emotion regulation. The first step in theory construction methodology (TCM) is to identify relevant phenomena (Borsboom et al., 2020). The present study takes that first step by mapping the relevant phenomena in this area of research using a relatively comprehensive and objective method.

An important direction for future research would be to formalize these inductive insights into a new overarching theory of adolescent emotion regulation. This would require describing and quantifying the nature of the relationship of each phenomenon in relation to adolescent emotion regulation (Borsboom et al., 2020). This involves abductive reasoning: the attribution of observations to causal explanatory principles (Haig, 2005). As a starting point for this effort, the co-occurrence graphs presented here could be used as templates for a nomological network: a proto-theoretical diagrammatic representation that describes causal relationships between relevant phenomena (see Alavi et al., 2018).

## Stengths and Limitations

One strength of the present study is that it was more comprehensive than previous reviews of the literature in two respects (cf. Bariola et al., 2011; Coe-Odess et al., 2019): First, in contrast to prior literature reviews, the present study was based on a systematic literature search. Second, prior reviews relied on narrative synthesis of a comparatively smaller number of publications, at most 241 (Coe-Odess et al., 2019). By contrast, the present study was able to map the literature more comprehensively, using text mining analysis to synthesize all 6305 identified records. Text mining offers unique advantages compared to narrative reviews (Littell, 2008), because it can cover vastly greater corpora than a sentient reader, and follows a somewhat more objective, transparent, and reproducible procedure. Both narrative and text mining reviews are initially labor-intensive. The key differences are that text mining systematic reviews are more scalable, because code can be applied to corpora of arbitrary size, and are easily updated when new literature is published. Once written, analysis code can be repurposed for reviews in different areas of research with relatively little effort. The present study illustrates that text mining systematic reviews extract relevant information from the published literature in a way that complements theory and narrative reviews. Moreover, text mining systematic reviews appear to be a suitable method for identifying relevant phenomena in a particular area of research. This can serve as the first step toward formal theory construction (Borsboom et al., 2020). The present study was the first to develop and implement this novel approach.

It should be noted that the present study has limitations as well. The key disadvantage of the method used is that these inductive text mining techniques are not able to extract *meaning* from the literature in the way a sentient reader would. This limitation is best addressed by considering the results as a starting point for further abductive reasoning and theory formation, deductive empirical research, or a narrative review focused on the emergent relevant phenomena. A related limitation is that the text mining methods used do not capture the nature of the relationship between co-occurring terms. Instead, terms were manually classified as potential causes, outcomes, protective factors, or indicators, based on domain knowledge. Efforts are underway to develop unsupervised algorithms capable of distilling implied causal links from scientific abstracts (An, Xiao, Yuan, Yang, & Alterovitz, 2019). Future research might substantially advance theory formation by applying such methods to the present corpus. Another limitation is that the present analyses were limited to keywords and abstracts. The primary obstacles to the analysis of full-text publications were limited access to articles behind paywalls, and the absence of a standardized Application Programming Interface (API) for the automatic retrieval of articles across publishing outlets. Comprehensive access to scientific publications is essential to all meta-scientific research, including narrative reviews and meta-analyses (e.g., to avoid bias in summary effect sizes). For text mining approaches in particular, the availability of large data sets is crucial. One example of a data set suitable for even more complex text mining analyses is a corpus of 400k+ full-text articles on COVID-19 that was made publicly available (L. L. Wang et al., 2020). Compiling such full-text data sets currently requires substantial manpower and financial resources, thus placing them out of reach for many researchers. Increased adoption of open access publishing, and the development of a unified API for article retrieval across publishing outlets, would facilitate curating full-text data sets. These changes would enable more widespread adoption and more informative application of meta-scientific (text mining) analyses. One final limitation is that several subjective decisions were made throughout the analysis process. This creative license is inherent to inductive studies (Wagenmakers et al., 2018), as there are infinite possible ways to conduct exploratory analyses. To address this limitation, subjective decisions are explicitly discussed throughout the manuscript, and all analysis code and data are made publicly available in a fully reproducible format (based on Van Lissa et al., 2020), so that others may explore alternative exploratory analysis strategies.

# Conclusion

There has been a call for the integration of the empirical literature on adolescent emotion regulation into an overarching framework. The first step towards this end is identifying relevant phenomena in this area of research. This article used a text mining systematic review to identify phenomena relevant to adolescents’ emotion regulation based on term frequency, and to map potential associations between phenomena based on term co-occurrence. The findings echoed several themes featured in the theoretical literature review, such as socialization and neurocognitive factors, which illustrates the validity of this new method for identifying relevant phenomena. Importantly, the findings also revealed undertheorized themes, such as developmental disorders, physical health, external stressors, structural disadvantage, substance use, identity, moral, and sexual development. This illustrates how text mining systematic reviews may complement narrative reviews. Several of the identified themes are particularly relevant in adolescence, either because they first emerge in this life phase (e.g., sexual development) or because they relate to age-graded developmental challenges (e.g., identity development). This reinforces the prior observation that general theories of emotion regulation cannot simply be generalized to adolescence, and emphasizes the importance of developing theory specific to adolescence. Future theoretical work might integrate these undertheorized themes into an overarching framework, and empirical research might consider them as promising areas for future research, or as potential confounders and contributing causes in research on adolescents’ emotion regulation.

# References

Alavi, M., Archibald, M., McMaster, R., Lopez, V., & Cleary, M. (2018). Aligning theory and methodology in mixed methods research: Before Design Theoretical Placement. *International Journal of Social Research Methodology*, *21*(5), 527–540. <https://doi.org/10.1080/13645579.2018.1435016>

Aldao, A., Nolen-Hoeksema, S., & Schweizer, S. (2010). Emotion-regulation strategies across psychopathology: A meta-analytic review. *Clinical Psychology Review*, *30*(2), 217–237.

An, N., Xiao, Y., Yuan, J., Yang, J., & Alterovitz, G. (2019). Extracting causal relations from the literature with word vector mapping. *Computers in Biology and Medicine*, *115*, 103524. <https://doi.org/10.1016/j.compbiomed.2019.103524>

Arnett, J. J. (1999). Adolescent storm and stress, reconsidered. *American Psychologist*, *54*(5), 317–326. <https://doi.org/10.1037/0003-066X.54.5.317>

Bariola, E., Gullone, E., & Hughes, E. K. (2011). Child and adolescent emotion regulation: The role of parental emotion regulation and expression. *Clinical Child and Family Psychology Review*, *14*(2), 198. <https://doi.org/10.1007/s10567-011-0092-5>

Bell, K. L., & Calkins, S. D. (2000). Relationships as Inputs and Outputs of Emotion Regulation. *Psychological Inquiry*, *11*(3), 160–163. Retrieved from <http://www.jstor.org/stable/1449794>

Benoit, K., Watanabe, K., Wang, H., Nulty, P., Obeng, A., Müller, S., & Matsuo, A. (2018). Quanteda: An R package for the quantitative analysis of textual data. *Journal of Open Source Software*, *3*(30), 774. <https://doi.org/10.21105/joss.00774>

Blei, D. M., Ng, A. Y., & Jordan, M. I. (2003). Latent Dirichlet Allocation. *Journal of Machine Learning Research*, *3*(Jan), 993–1022. Retrieved from <http://www.jmlr.org/papers/v3/blei03a>

Borsboom, D., Dalege, J., Kievit, R. A., & Haig, B. D. (2020, February 29). Theory Construction Methodology: A practical framework for building theories in psychology. Retrieved from <https://doi.org/10.31234/osf.io/w5tp8>

Braet, C., Theuwis, L., Durme, K. V., Vandewalle, J., Vandevivere, E., Wante, L., … Goossens, L. (2014). Emotion regulation in children with emotional problems. *Cognitive Therapy and Research*, *38*(5), 493–504. <https://doi.org/10.1007/s10608-014-9616-x>

Bronfenbrenner, U., & Morris, P. A. (2007). The bioecological model of human development. In *Handbook of Child Psychology*. John Wiley & Sons, Inc. <https://doi.org/10.1002/9780470147658.chpsy0114>

Brown, L. K., Tolou-Shams, M., Lescano, C., Houck, C., Zeidman, J., Pugatch, D., & Lourie, K. J. (2006). Depressive Symptoms as a Predictor of Sexual Risk among African American Adolescents and Young Adults. *Journal of Adolescent Health*, *39*(3), 444.e1–444.e8. <https://doi.org/10.1016/j.jadohealth.2006.01.015>

Buss, K. A., Cole, P. M., & Zhou, A. M. (2019). Theories of Emotional Development: Where Have We Been and Where Are We Now? In V. LoBue, K. Pérez-Edgar, & K. A. Buss (Eds.), *Handbook of Emotional Development* (pp. 7–25). Cham: Springer International Publishing. <https://doi.org/10.1007/978-3-030-17332-6_2>

Campbell, S. M., Zimmer-Gembeck, M. J., & Duffy, A. (2019). Friends and Education: Identity Patterns across Domains and Associations with Emotion Dysregulation and Identity Disturbance. *Journal of Youth and Adolescence*, *48*(4), 703–716. <https://doi.org/10.1007/s10964-018-0924-4>

Cappadocia, M. C., Weiss, J. A., & Pepler, D. (2012). Bullying Experiences Among Children and Youth with Autism Spectrum Disorders. *Journal of Autism and Developmental Disorders*, *42*(2), 266–277. <https://doi.org/10.1007/s10803-011-1241-x>

Coe-Odess, S. J., Narr, R. K., & Allen, J. P. (2019). Emergent Emotions in Adolescence. In V. LoBue, K. Pérez-Edgar, & K. A. Buss (Eds.), *Handbook of Emotional Development* (pp. 595–625). Cham: Springer International Publishing. <https://doi.org/10.1007/978-3-030-17332-6_23>

Cracco, E., Goossens, L., & Braet, C. (2017). Emotion regulation across childhood and adolescence: Evidence for a maladaptive shift in adolescence. *European Child & Adolescent Psychiatry*, *26*(8), 909–921. <https://doi.org/10.1007/s00787-017-0952-8>

Crone, E. A., & Dahl, R. E. (2012). Understanding adolescence as a period of social–affective engagement and goal flexibility. *Nature Reviews Neuroscience*, *13*(9), 636–650. <https://doi.org/10.1038/nrn3313>

Gross, J. J. (2013). *Handbook of emotion regulation*. Guilford publications.

Haig, B. D. (2005). Exploratory Factor Analysis, Theory Generation, and Scientific Method. *Multivariate Behavioral Research*, *40*(3), 303–329. <https://doi.org/10.1207/s15327906mbr4003_2>

Holodynski, M., & Friedlmeier, W. (2006). *Development of Emotions and Emotion Regulation*. Springer Science & Business Media. Retrieved from <http://books.google.com?id=OvJHncpZEQUC>

Lapsley, D. (2015). Moral Identity and Developmental Theory. *Human Development*, *58*(3), 164–171. <https://doi.org/10.1159/000435926>

Lee, F. S., Heimer, H., Giedd, J. N., Lein, E. S., Šestan, N., Weinberger, D. R., & Casey, B. J. (2014). Adolescent mental health—Opportunity and obligation. *Science*, *346*(6209), 547–549. <https://doi.org/10.1126/science.1260497>

Lemerise, E. A., & Arsenio, W. F. (2000). An Integrated Model of Emotion Processes and Cognition in Social Information Processing. *Child Development*, *71*(1), 107–118. <https://doi.org/10.1111/1467-8624.00124>

Littell, J. H. (2008). Evidence-based or biased? The quality of published reviews of evidence-based practices. *Children and Youth Services Review*, *30*(11), 1299–1317. <https://doi.org/10.1016/j.childyouth.2008.04.001>

Martin, F., & Johnson, M. (2015). More Efﬁcient Topic Modelling Through a Noun Only Approach. In *Proceedings of Australasian Language Technology Association Workshop* (pp. 111–115).

Meeus, W. (2011). The Study of Adolescent Identity Formation 2000-2010: A Review of Longitudinal Research. *Journal of Research on Adolescence*, *21*(1), 75–94. <https://doi.org/10.1111/j.1532-7795.2010.00716.x>

Morris, A. S., Silk, J. S., Steinberg, L., Myers, S. S., & Robinson, L. R. (2007). The role of the family context in the development of emotion regulation. *Social Development*, *16*(2), 361–388. <https://doi.org/10.1111/j.1467-9507.2007.00389.x>

Myers, H. F. (2009). Ethnicity- and socio-economic status-related stresses in context: An integrative review and conceptual model. *Journal of Behavioral Medicine*, *32*(1), 9–19. <https://doi.org/10.1007/s10865-008-9181-4>

Ouzzani, M., Hammady, H., Fedorowicz, Z., & Elmagarmid, A. (2016). Rayyan—a web and mobile app for systematic reviews. *Systematic Reviews*, *5*(1), 210. <https://doi.org/10.1186/s13643-016-0384-4>

Pierrehumbert, B., Bader, M., Miljkovitch, R., Mazet, P., Amar, M., & Halfon, O. (2002). Strategies of emotion regulation in adolescents and young adults with substance dependence or eating disorders. *Clinical Psychology & Psychotherapy*, *9*(6), 384–394. <https://doi.org/10.1002/cpp.339>

Pleck, J. (2004). Paternal involvement: Revised conceptualization and theoretical linkages with child outcomes. In M. E. Lamb (Ed.), *The role of the father in child development* (pp. 58–93). Hoboken, New Jersey: John Wiley & Sons.

Porges, S. W. (1995). Orienting in a defensive world: Mammalian modifications of our evolutionary heritage. A Polyvagal Theory. *Psychophysiology*, *32*(4), 301–318. <https://doi.org/10.1111/j.1469-8986.1995.tb01213.x>

Reindl, M., Gniewosz, B., & Reinders, H. (2016). Socialization of emotion regulation strategies through friends. *Journal of Adolescence*, *49*, 146–157. <https://doi.org/10.1016/j.adolescence.2016.03.008>

Riediger, M., & Klipker, K. (2014). Emotion regulation in adolescence. In *Handbook of emotion regulation* (Vols. 1–Book, Section, pp. 187–202). Guilford Press.

Sameroff, A. (2010). A unified theory of development: A dialectic integration of nature and nurture. *Child Development*, *81*(1), 6–22. <https://doi.org/10.1111/j.1467-8624.2009.01378.x>

Spithoven, A. W. M., Bijttebier, P., & Goossens, L. (2017). It is all in their mind: A review on information processing bias in lonely individuals. *Clinical Psychology Review*, *58*, 97–114. <https://doi.org/10.1016/j.cpr.2017.10.003>

Sroufe, L. A. (1995). *Emotional Development: The Organization of Emotional Life in the Early Years*. Cambridge: Cambridge University Press. <https://doi.org/10.1017/CBO9780511527661>

Staaks, J. (2019). Systematic Review Search Support. Retrieved March 23, 2020, from <https://doi.org/10.17605/OSF.IO/49T8X>

Steinberg, L. (2014). *Age of Opportunity: Lessons from the New Science of Adolescence*. Houghton Mifflin Harcourt. Retrieved from <http://books.google.com?id=3cubAwAAQBAJ>

Stifter, C., & Augustine, M. (2019). Emotion Regulation. In V. LoBue, K. Pérez-Edgar, & K. A. Buss (Eds.), *Handbook of Emotional Development* (pp. 405–430). Cham: Springer International Publishing. <https://doi.org/10.1007/978-3-030-17332-6_16>

Stikkelbroek, Y., Bodden, D. H. M., Kleinjan, M., Reijnders, M., & Baar, A. L. van. (2016). Adolescent Depression and Negative Life Events, the Mediating Role of Cognitive Emotion Regulation. *PLOS ONE*, *11*(8), e0161062. <https://doi.org/10.1371/journal.pone.0161062>

Van de Schoot, R., De Bruin, J., Schram, R., Zahedi, P., De Boer, J., Weijdema, F., … Oberski, D. (2020). ASReview: Active learning for systematic reviews (Version v0.9.5). Zenodo. Retrieved from <https://doi.org/10.5281/zenodo.3828293>

Van Lissa, C. J., Brandmaier, A. M., Brinkman, L., Lamprecht, A.-L., Peikert, A., Struiksma, M. E., & Vreede, B. (2020). WORCS: A Workflow for Open Reproducible Code in Science. <https://doi.org/10.17605/OSF.IO/ZCVBS>

Van Lissa, C. J., Hawk, S. T., de Wied, M., Koot, H. M., van Lier, P., & Meeus, W. (2014). The longitudinal interplay of affective and cognitive empathy within and between adolescents and mothers. *Developmental Psychology*, *50*(4), 1219–1225. <https://doi.org/10.1037/a0035050>

Van Lissa, C. J., Hawk, S. T., Koot, H. M., Branje, S., & Meeus, W. H. J. (2017). The cost of empathy: Parent-adolescent conflict predicts emotion dysregulation for highly empathic youth. *Developmental Psychology*, *ePub*(ePub), ePub. <https://doi.org/10.1037/dev0000361>

Van Lissa, C. J., & Keizer, R. (2020). Mothers’ and fathers’ quantitative and qualitative parenting in relation to children’s emotional adjustment: A between- and within-family investigation. *Developmental Psychology*, *in press*.

Van Lissa, C. J., Keizer, R., Van Lier, P. A. C., Meeus, W. H. J., & Branje, S. (2019). The role of fathers’ versus mothers’ parenting in emotion-regulation development from mid–late adolescence: Disentangling between-family differences from within-family effects. *Developmental Psychology*, *55*(2), 377–389. <https://doi.org/10.1037/dev0000612>

Vrolijk, P., Van Lissa, C. J., Branje, S. J. T., Meeus, W. H. J., & Keizer, R. (2020). Longitudinal Linkages Between Father and Mother Autonomy Support and Adolescent Problem Behaviors: Between-Family Differences and Within-Family Effects. *Journal of Youth and Adolescence*, *49*(11), 2372–2387. <https://doi.org/10.1007/s10964-020-01309-8>

Wagenmakers, E.-J., Dutilh, G., & Sarafoglou, A. (2018). The Creativity-Verification Cycle in Psychological Science: New Methods to Combat Old Idols. *Perspectives on Psychological Science*, *13*(4), 418–427. <https://doi.org/10.1177/1745691618771357>

Wang, L. L., Lo, K., Chandrasekhar, Y., Reas, R., Yang, J., Eide, D., … Kohlmeier, S. (2020). CORD-19: The Covid-19 Open Research Dataset. *ArXiv*. Retrieved from <https://www.ncbi.nlm.nih.gov/pmc/articles/PMC7251955/>

Wang, Y., Hawk, S. T., & Zong, W. (2020). Bidirectional effects between expressive regulatory abilities and peer acceptance among Chinese adolescents. *Journal of Experimental Child Psychology*, *199*, 104891. <https://doi.org/10.1016/j.jecp.2020.104891>

Wijffels, J. (2019). *Textrank: Summarize text by ranking sentences and finding keywords*. Retrieved from <https://CRAN.R-project.org/package=textrank>

Zimmermann, P., & Iwanski, A. (2014). Emotion regulation from early adolescence to emerging adulthood and middle adulthood: Age differences, gender differences, and emotion-specific developmental variations. *International Journal of Behavioral Development*, *38*(2), 182–194.


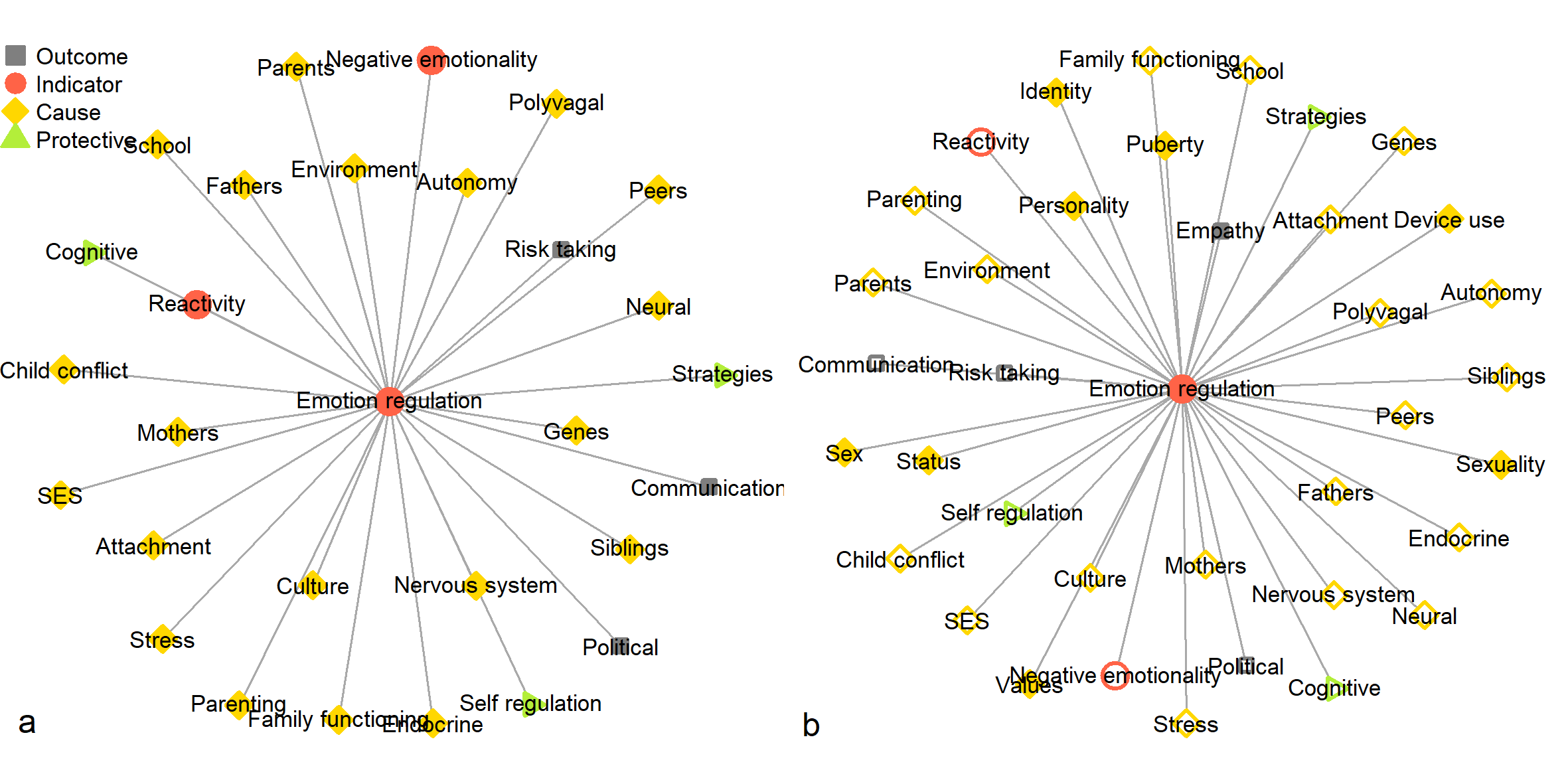


*Figure* *1:*. Phenomena relevant to adolescents’ emotion regulation according to theory (a) and narrative reviews (b; transparent nodes indicate constructs also present in the theory).


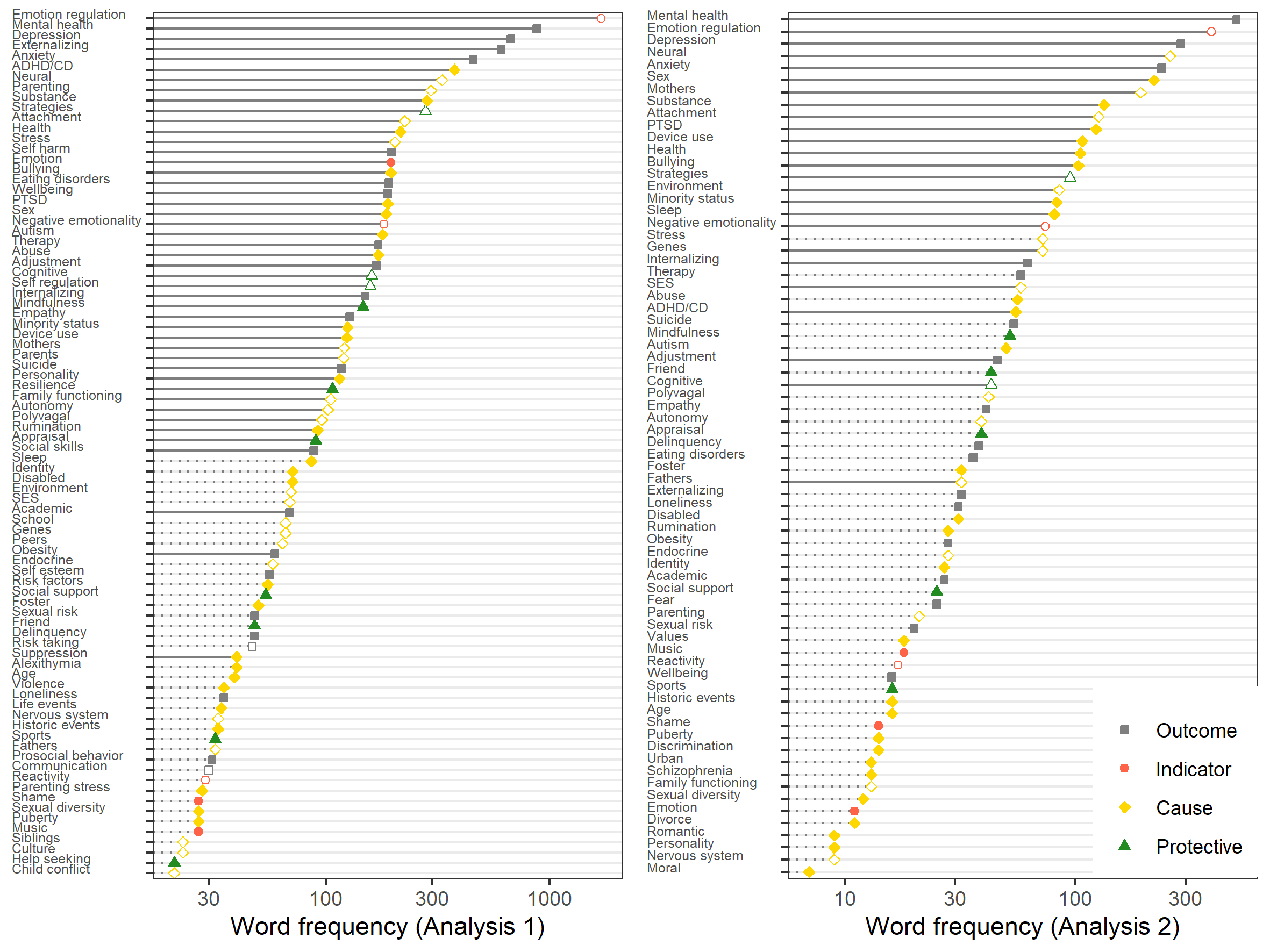


*Figure* *2:*. Frequency of terms in Analysis 1 and 2. Transparent nodes are constructs represented in theory (Figure 1), dotted lines are constructs absent from the co-occurrence graph.


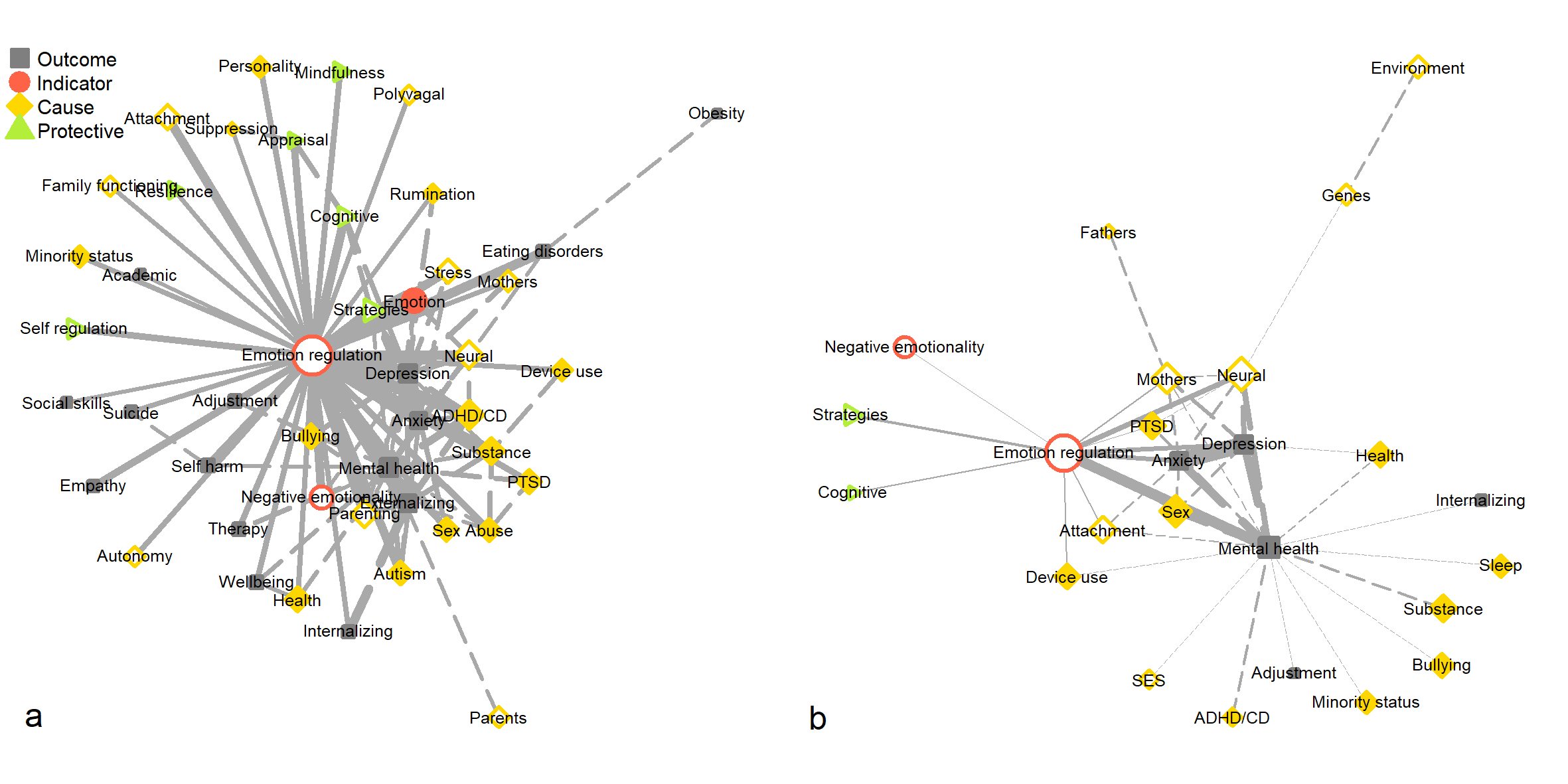


*Figure* *3:*. Map of phenomena relevant to adolescents’ emotion regulation based on term co-occurrence in author keywords (a) and abstracts (b). Size of lines and nodes represents frequency. Dashed lines represent links not involving emotion regulation. Transparent nodes indicate constructs also represented in the theoretical review.
