# Supplementary figures and images for: Mapping Phenomena Relevant to Adolescent Emotion Regulation: A Text-Mining Systematic Review
Source: Adolesc Res Rev. 2021 May 21;7(1):127–39. doi: 10.1007/s40894-021-00160-7 (PMC8138847; doi:10.1007/s40894-021-00160-7)

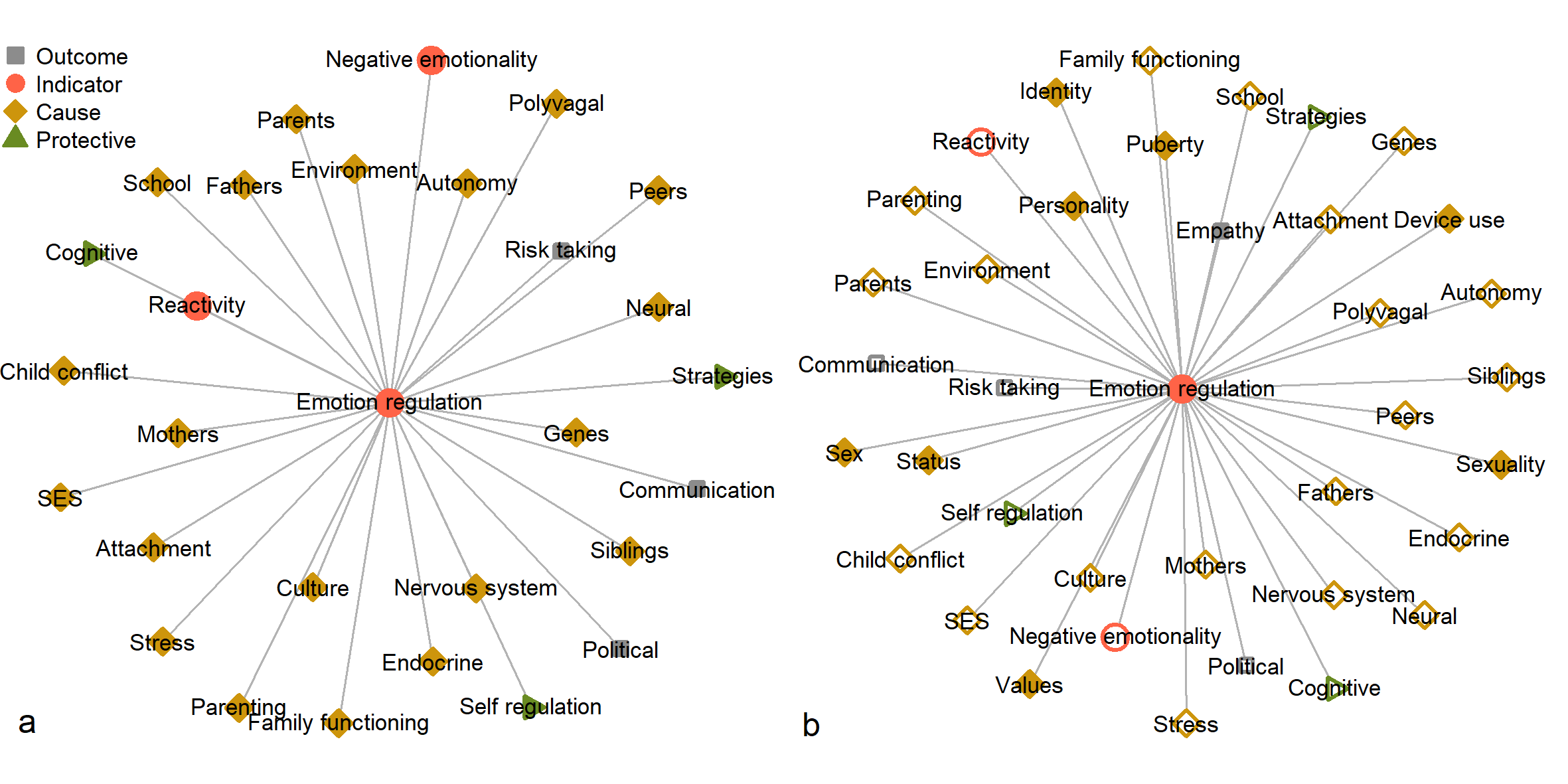

Supplement: Supplementary file 1 — Supplementary file1 (ZIP 54379 kb) [file 40894_2021_160_MOESM1_ESM.zip › veni_sysrev-master/baseline_network.png]

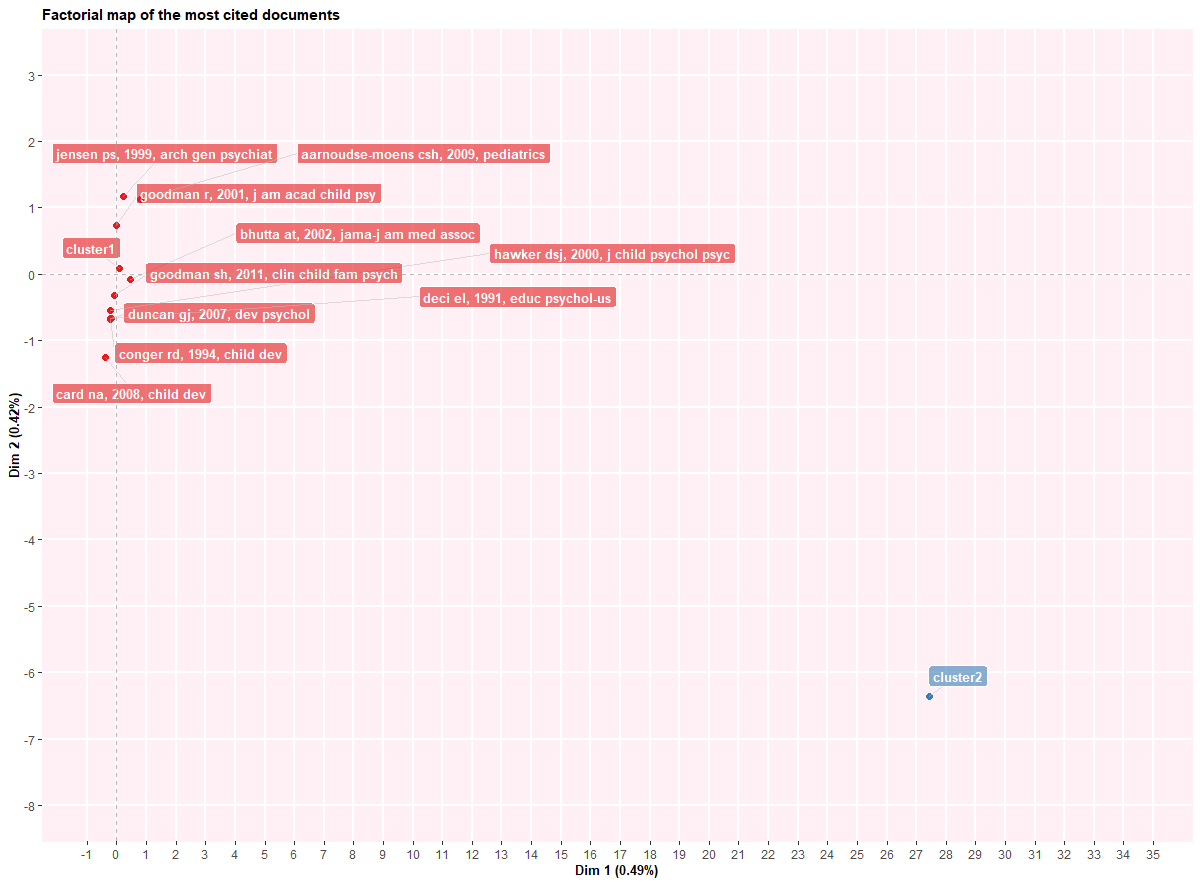

Supplement: Supplementary file 1 — Supplementary file1 (ZIP 54379 kb) [file 40894_2021_160_MOESM1_ESM.zip › veni_sysrev-master/factorial_maps]

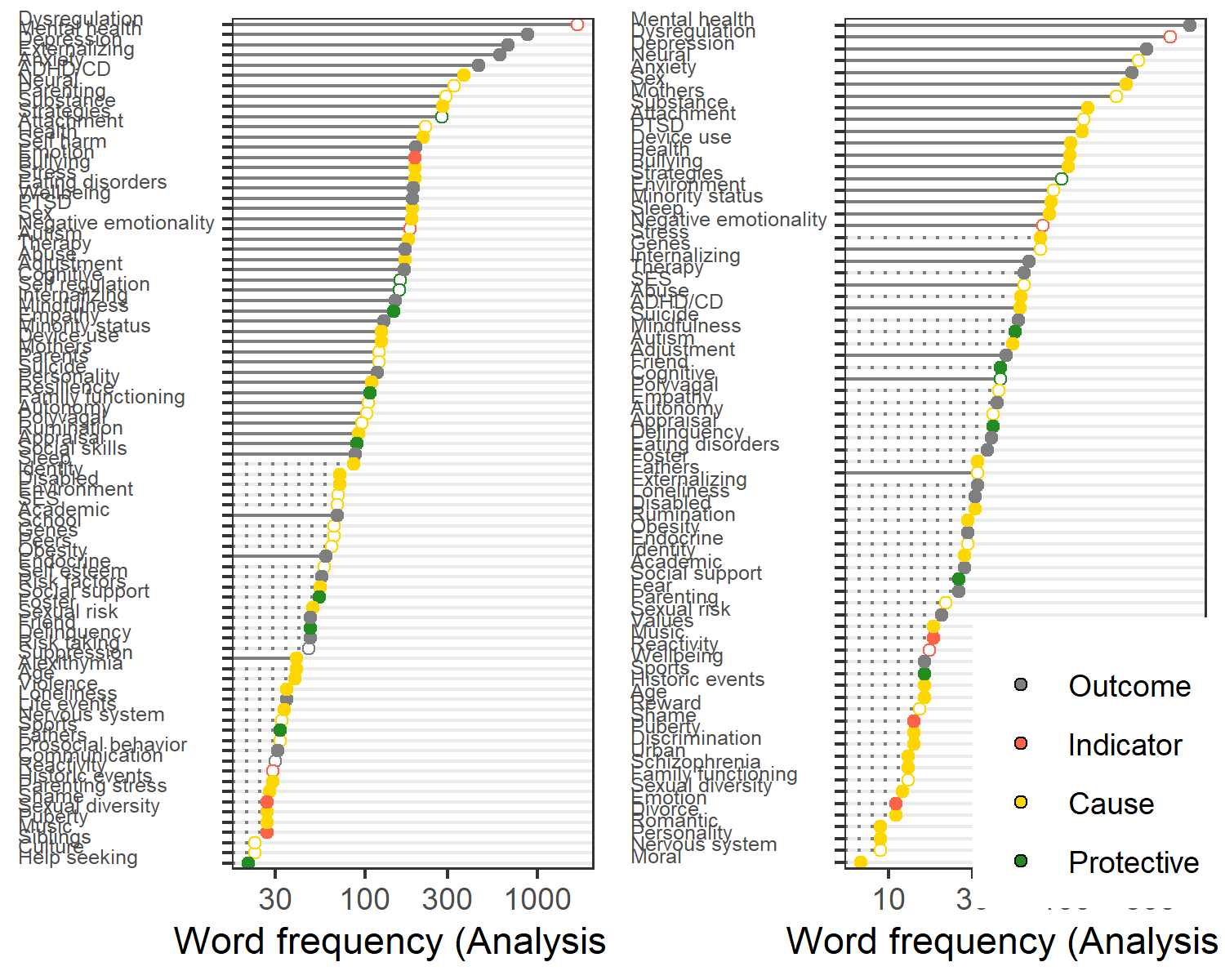

Supplement: Supplementary file 1 — Supplementary file1 (ZIP 54379 kb) [file 40894_2021_160_MOESM1_ESM.zip › veni_sysrev-master/manuscript_files/figure-docx/baseline-1.png]

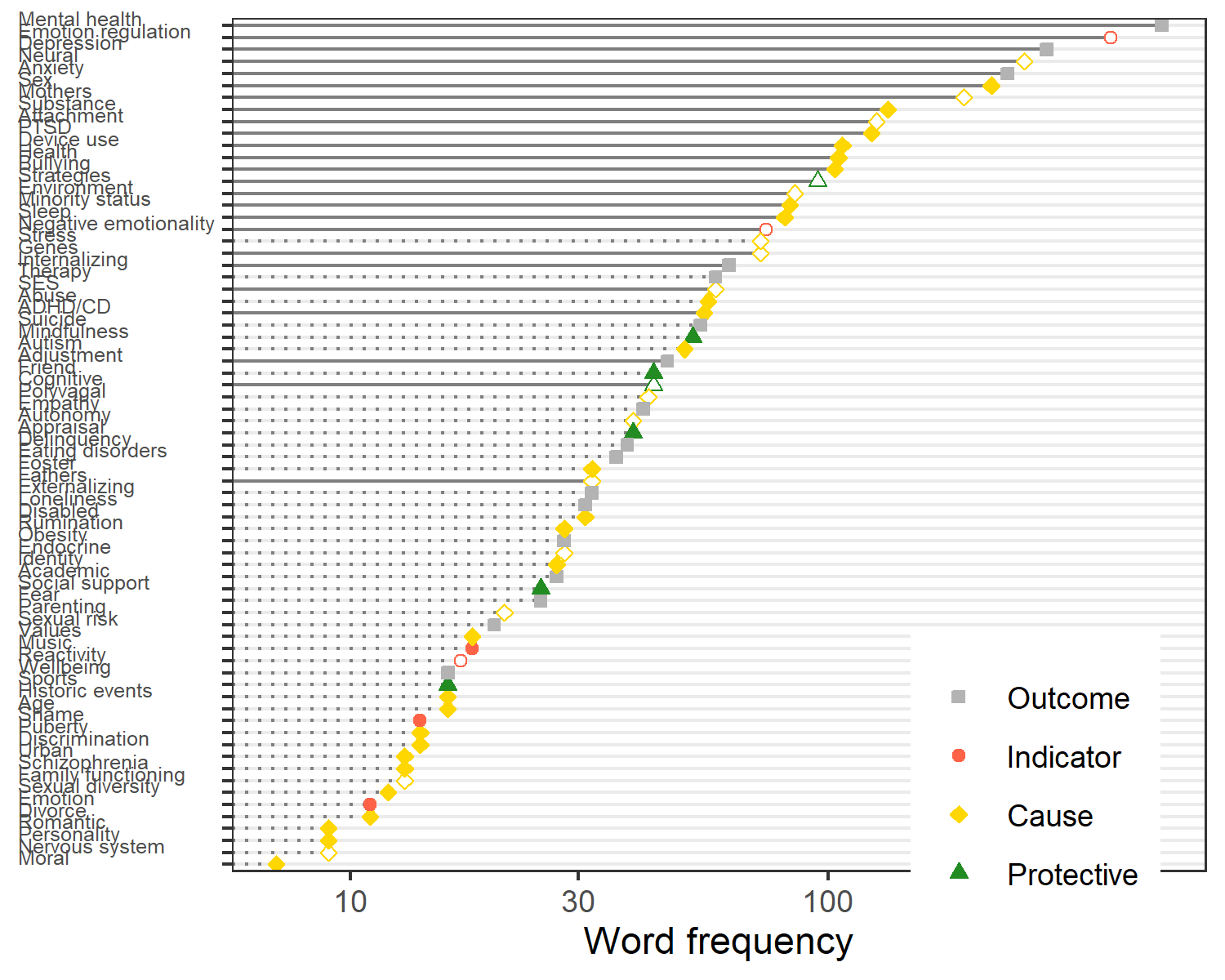

Supplement: Supplementary file 1 — Supplementary file1 (ZIP 54379 kb) [file 40894_2021_160_MOESM1_ESM.zip › veni_sysrev-master/manuscript_files/figure-docx/baselinecode-1.png]

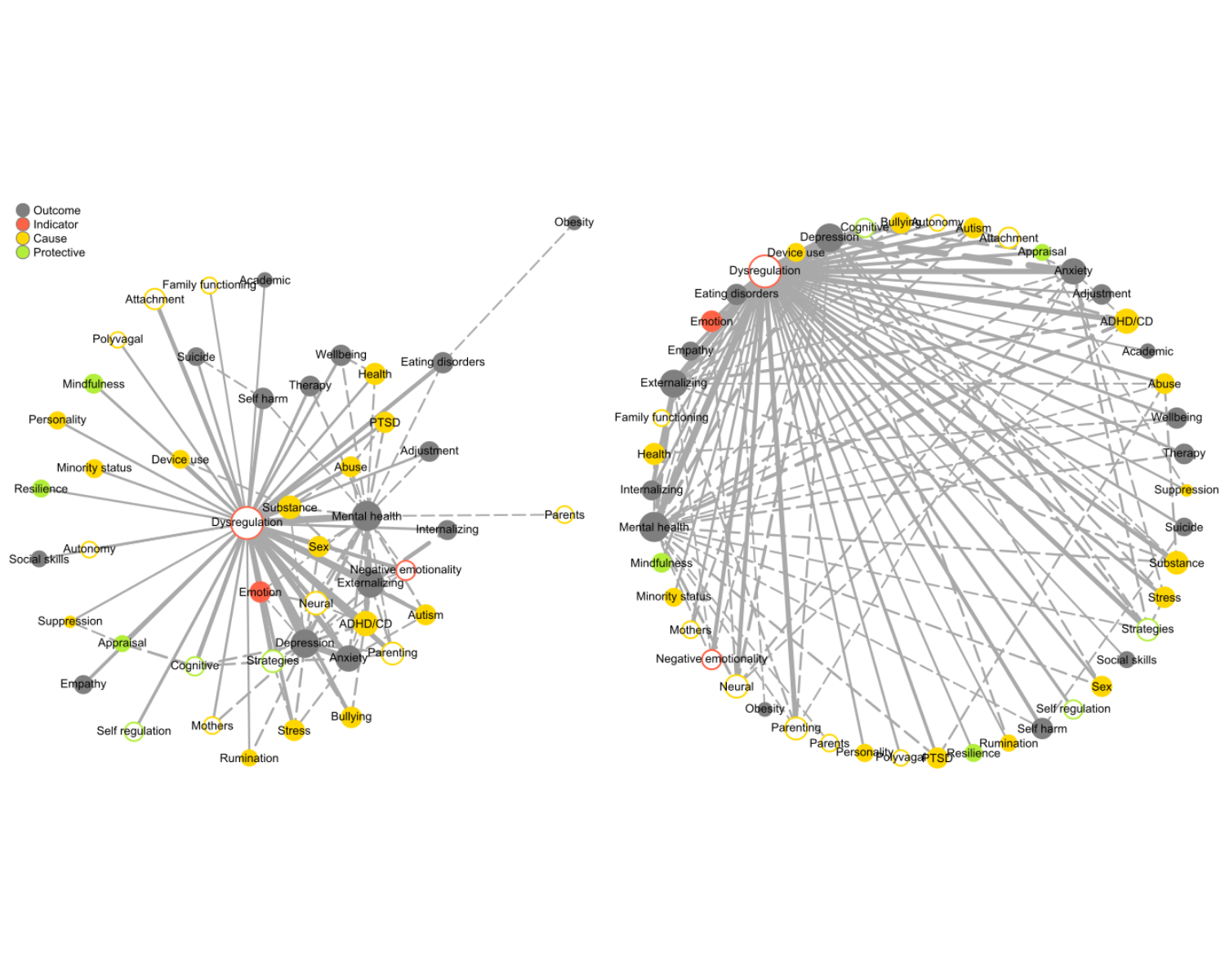

Supplement: Supplementary file 1 — Supplementary file1 (ZIP 54379 kb) [file 40894_2021_160_MOESM1_ESM.zip › veni_sysrev-master/manuscript_files/figure-docx/study1network-1.png]

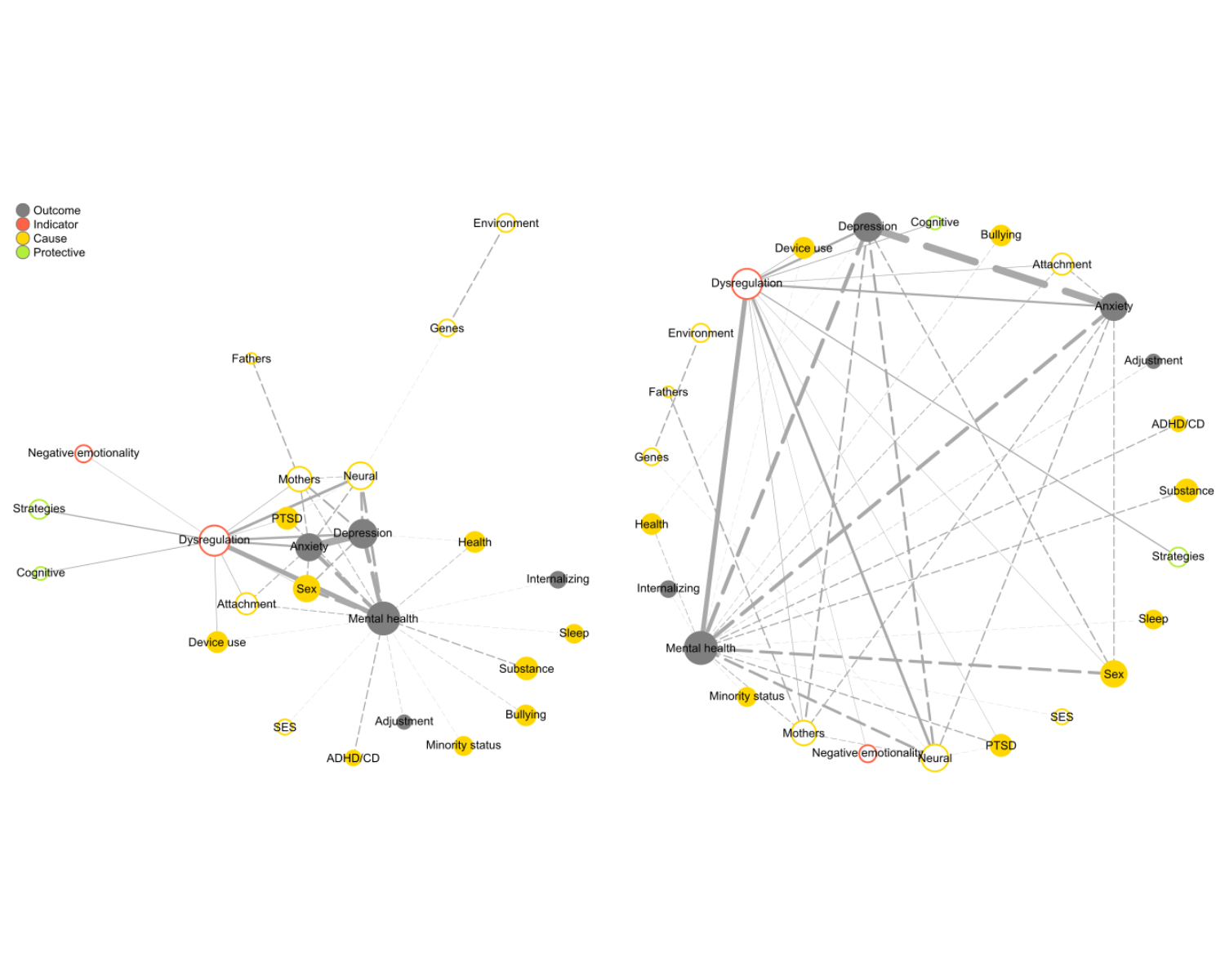

Supplement: Supplementary file 1 — Supplementary file1 (ZIP 54379 kb) [file 40894_2021_160_MOESM1_ESM.zip › veni_sysrev-master/manuscript_files/figure-docx/study2network-1.png]

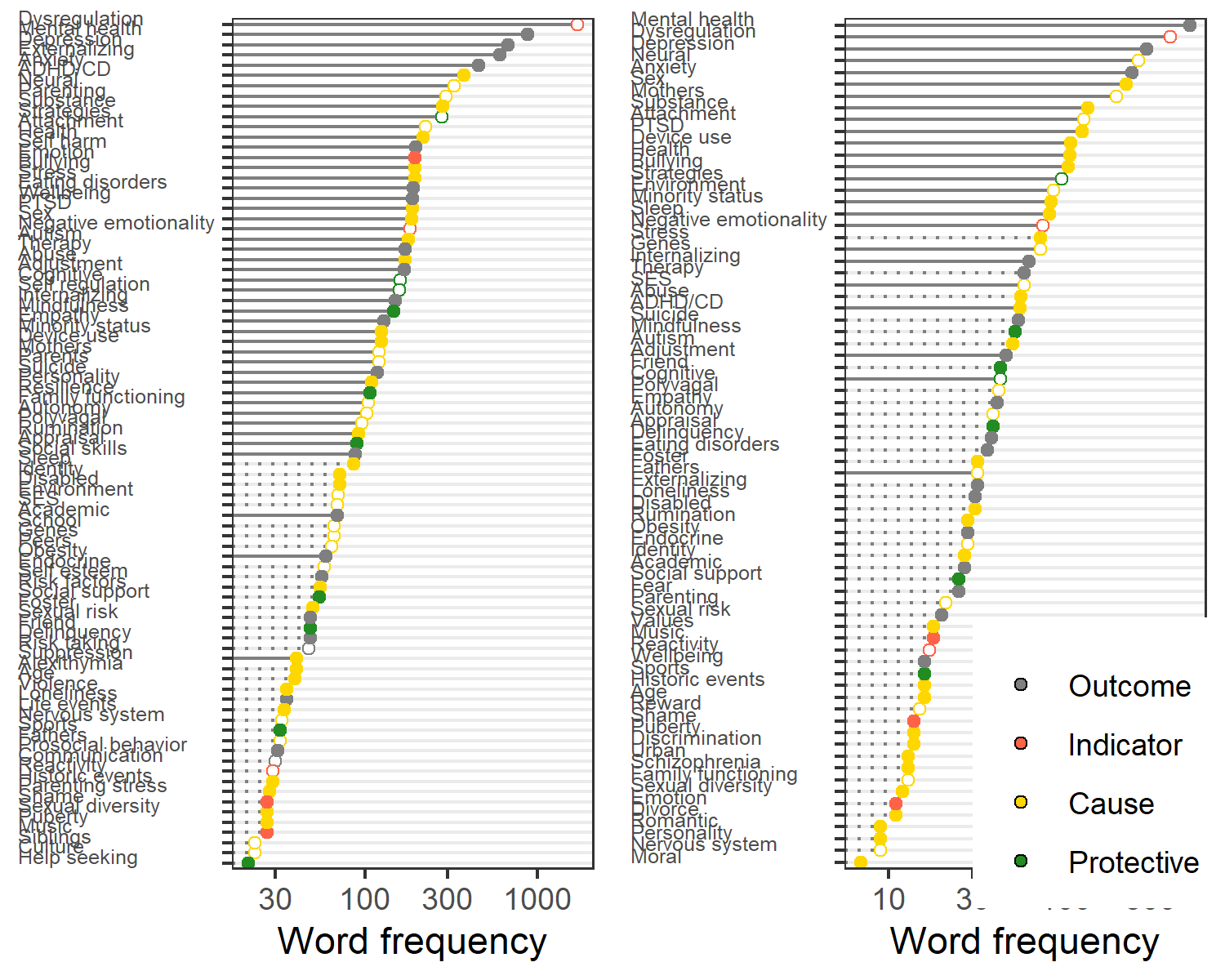

Supplement: Supplementary file 1 — Supplementary file1 (ZIP 54379 kb) [file 40894_2021_160_MOESM1_ESM.zip › veni_sysrev-master/manuscript_files/figure-docx/unnamed-chunk-33-1.png]

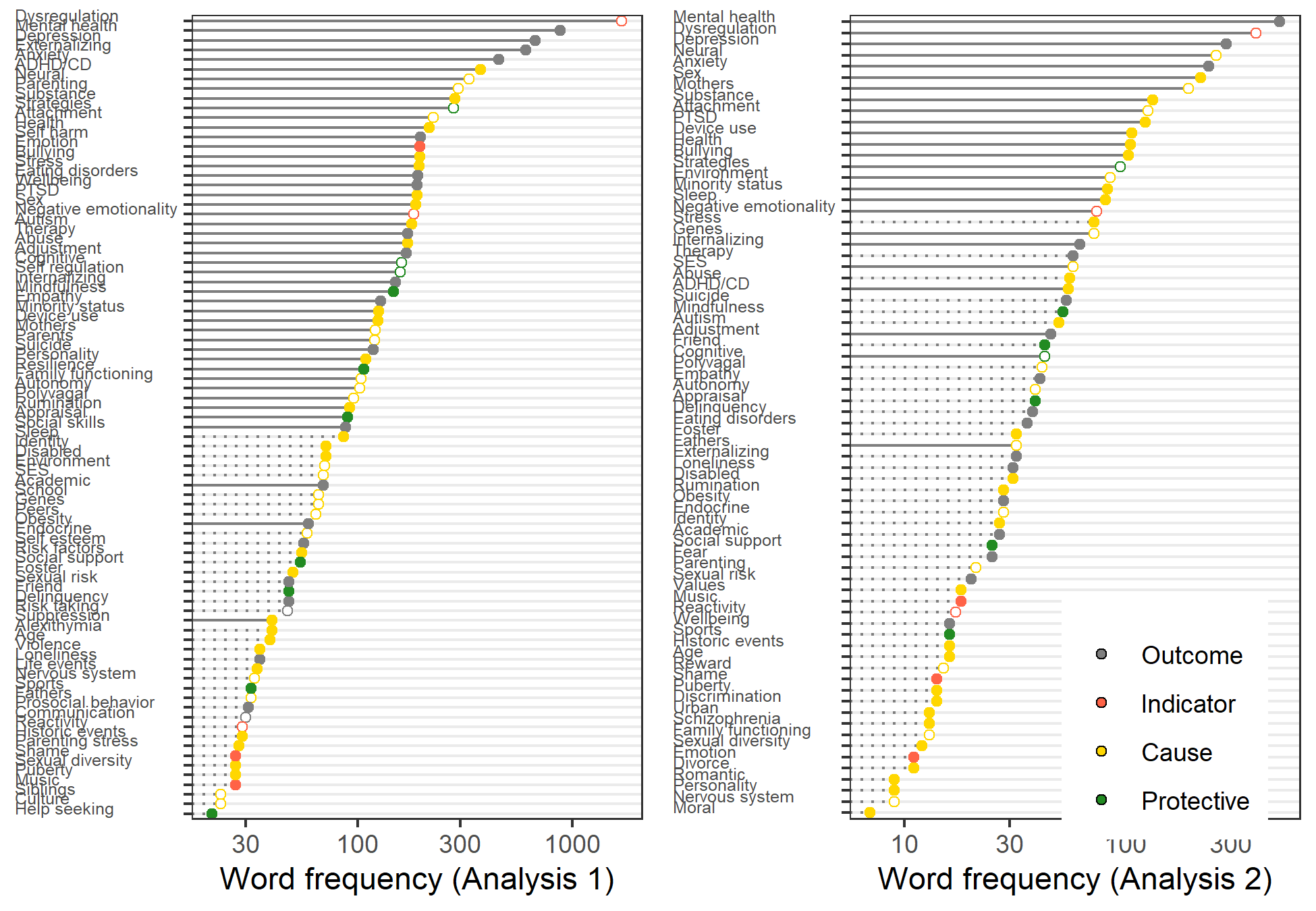

Supplement: Supplementary file 1 — Supplementary file1 (ZIP 54379 kb) [file 40894_2021_160_MOESM1_ESM.zip › veni_sysrev-master/manuscript_files/figure-latex/baseline-1.png]

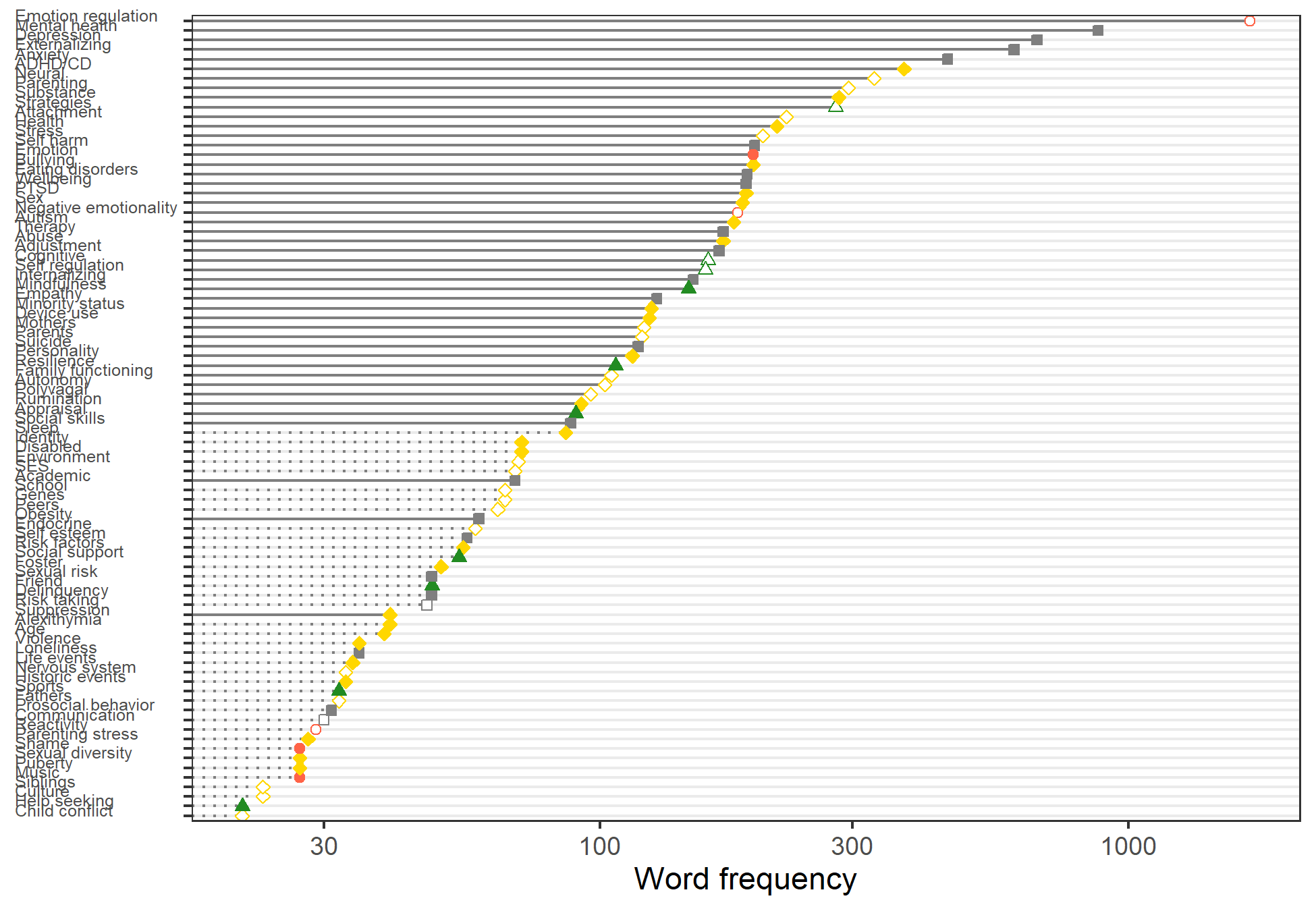

Supplement: Supplementary file 1 — Supplementary file1 (ZIP 54379 kb) [file 40894_2021_160_MOESM1_ESM.zip › veni_sysrev-master/manuscript_files/figure-latex/mergenetworks-1.png]

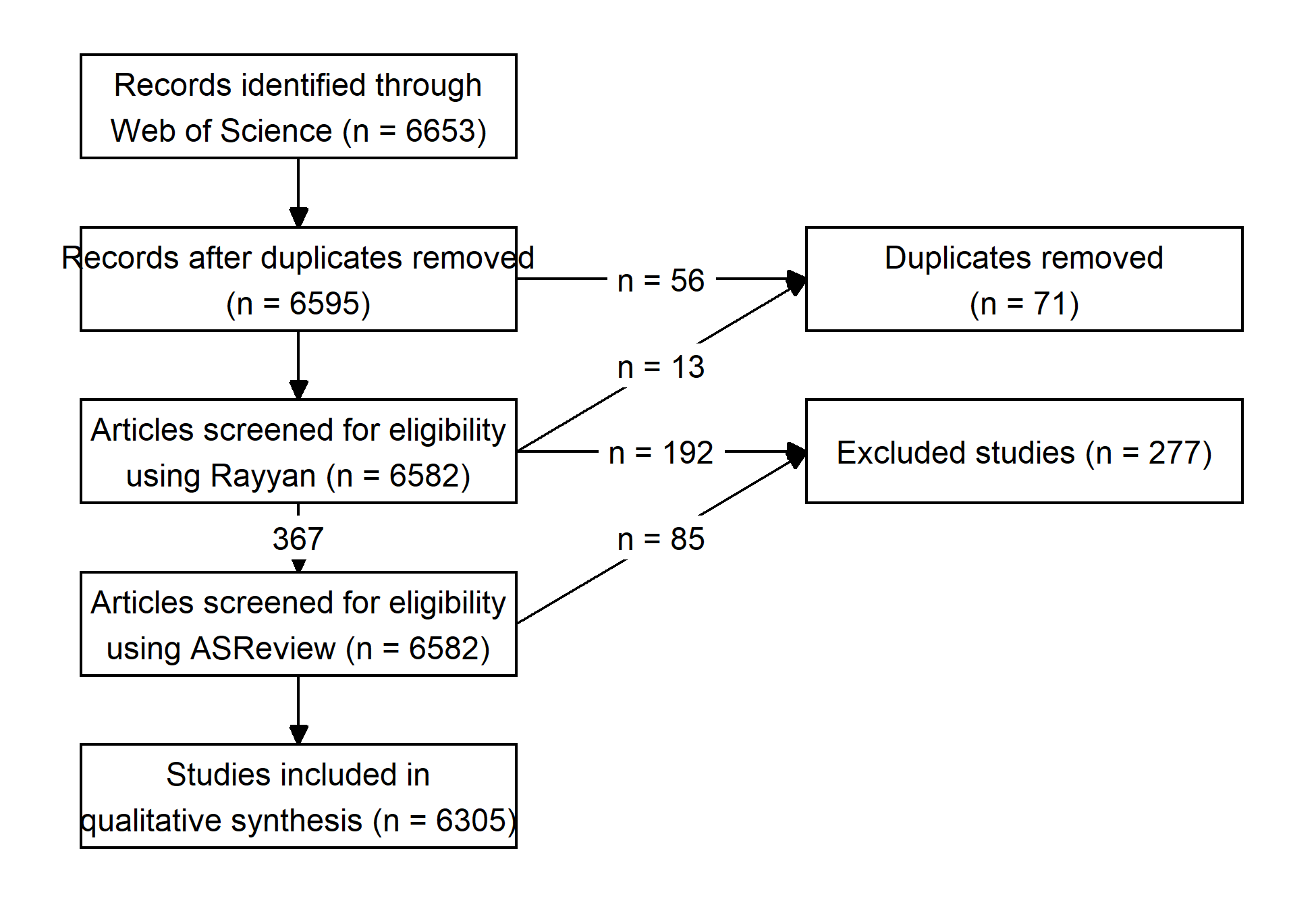

Supplement: Supplementary file 1 — Supplementary file1 (ZIP 54379 kb) [file 40894_2021_160_MOESM1_ESM.zip › veni_sysrev-master/manuscript_files/figure-latex/prismachart-1.png]

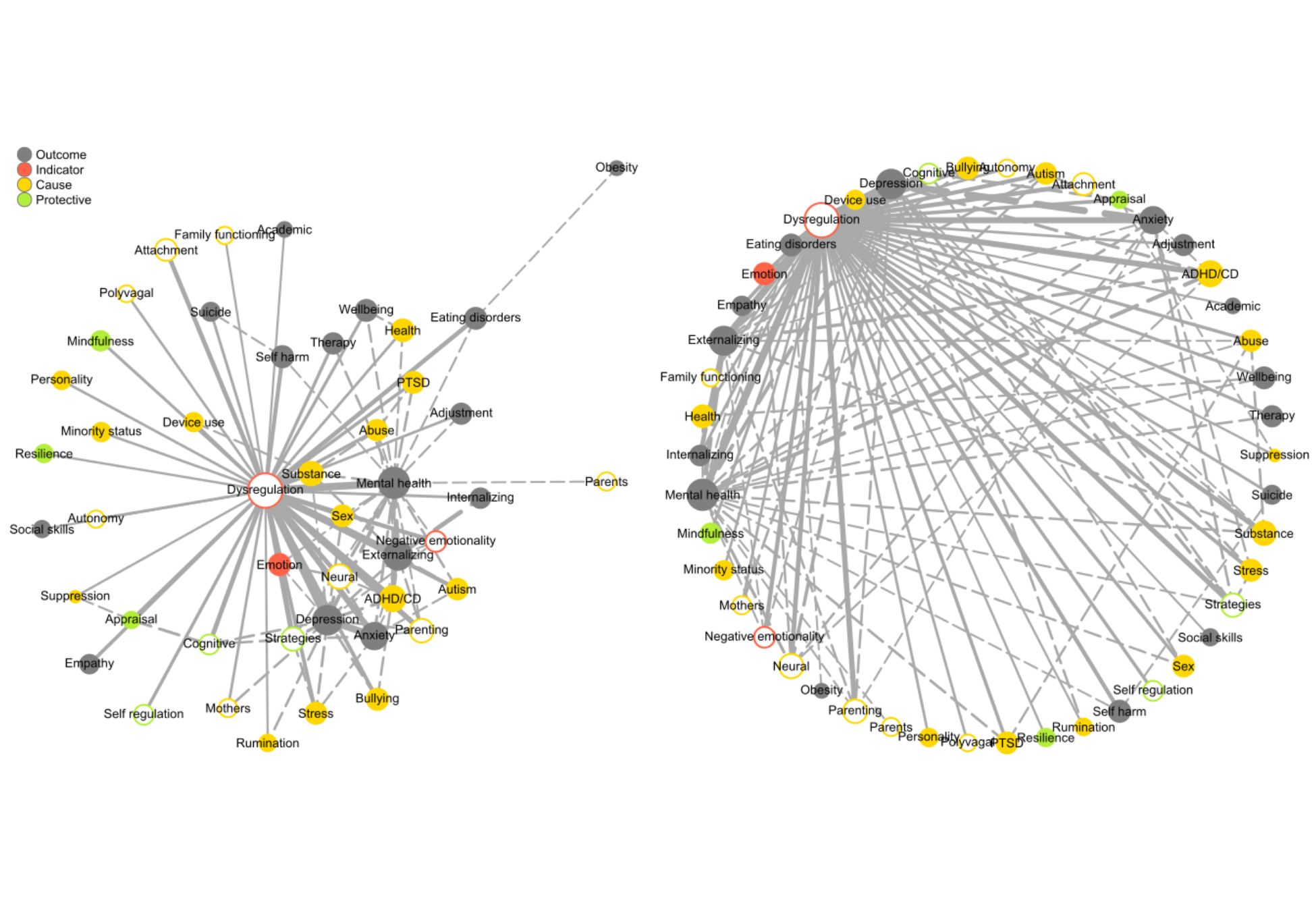

Supplement: Supplementary file 1 — Supplementary file1 (ZIP 54379 kb) [file 40894_2021_160_MOESM1_ESM.zip › veni_sysrev-master/manuscript_files/figure-latex/study1network-1.png]

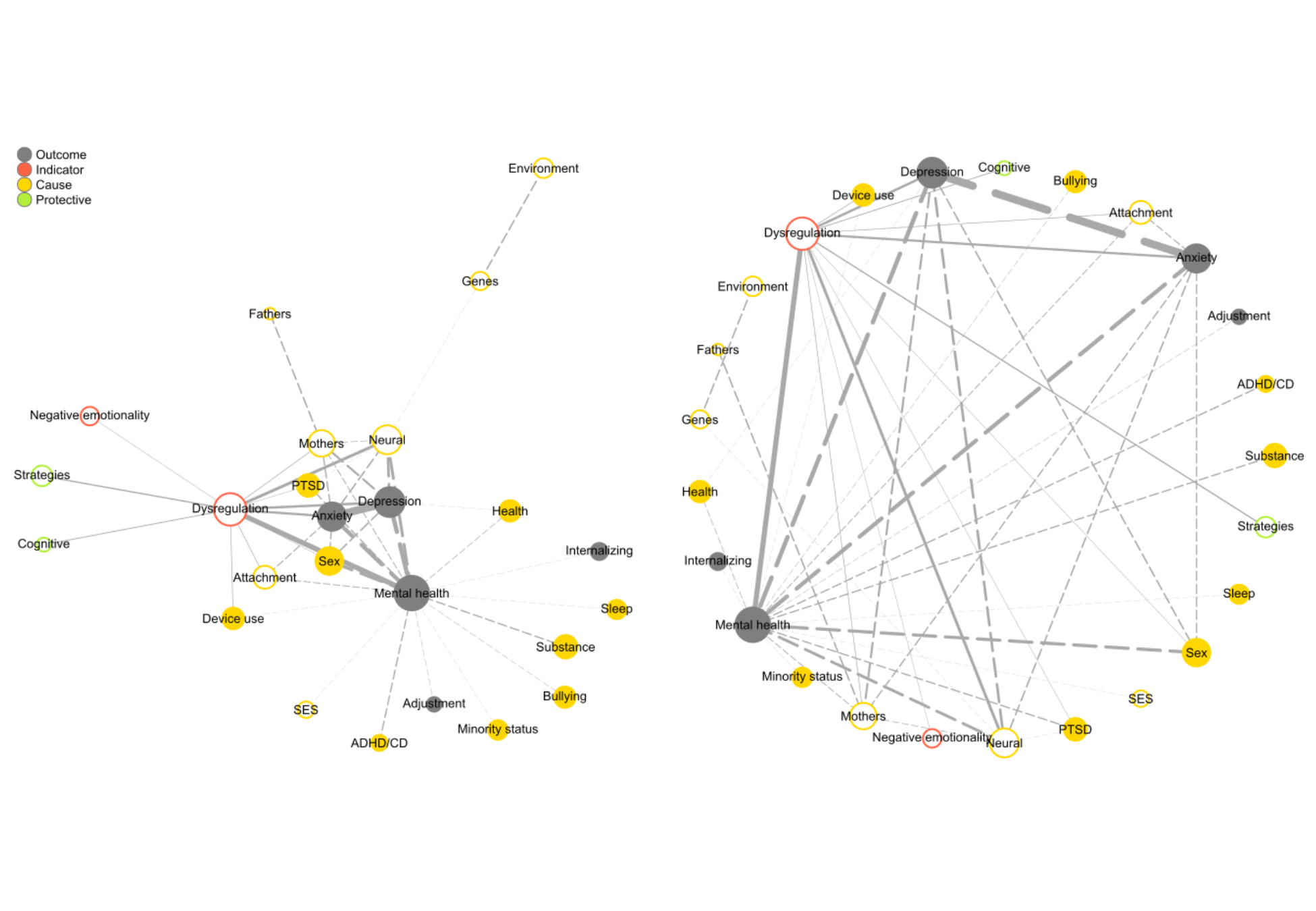

Supplement: Supplementary file 1 — Supplementary file1 (ZIP 54379 kb) [file 40894_2021_160_MOESM1_ESM.zip › veni_sysrev-master/manuscript_files/figure-latex/study2network-1.png]

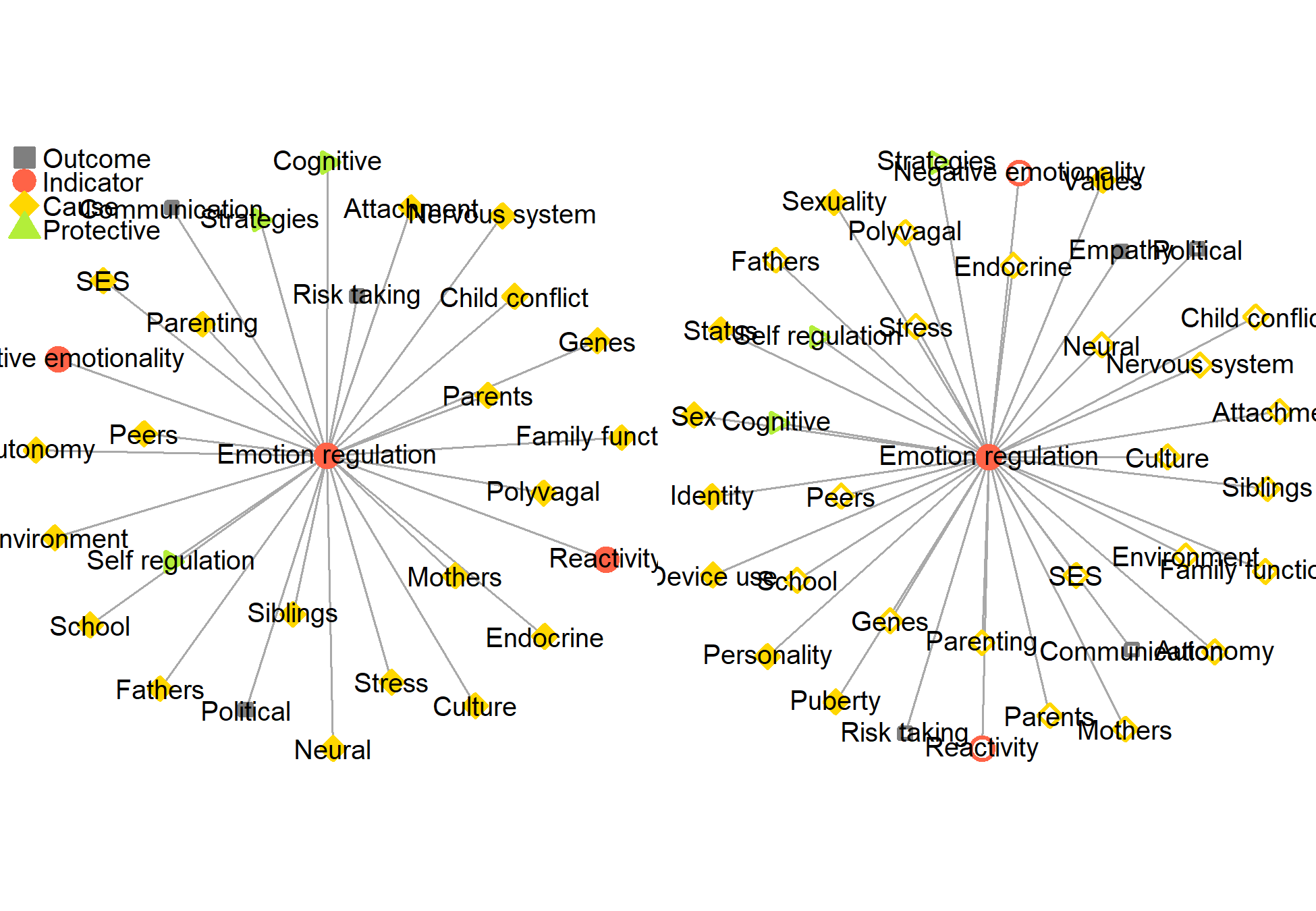

Supplement: Supplementary file 1 — Supplementary file1 (ZIP 54379 kb) [file 40894_2021_160_MOESM1_ESM.zip › veni_sysrev-master/manuscript_files/figure-latex/unnamed-chunk-2-1.png]

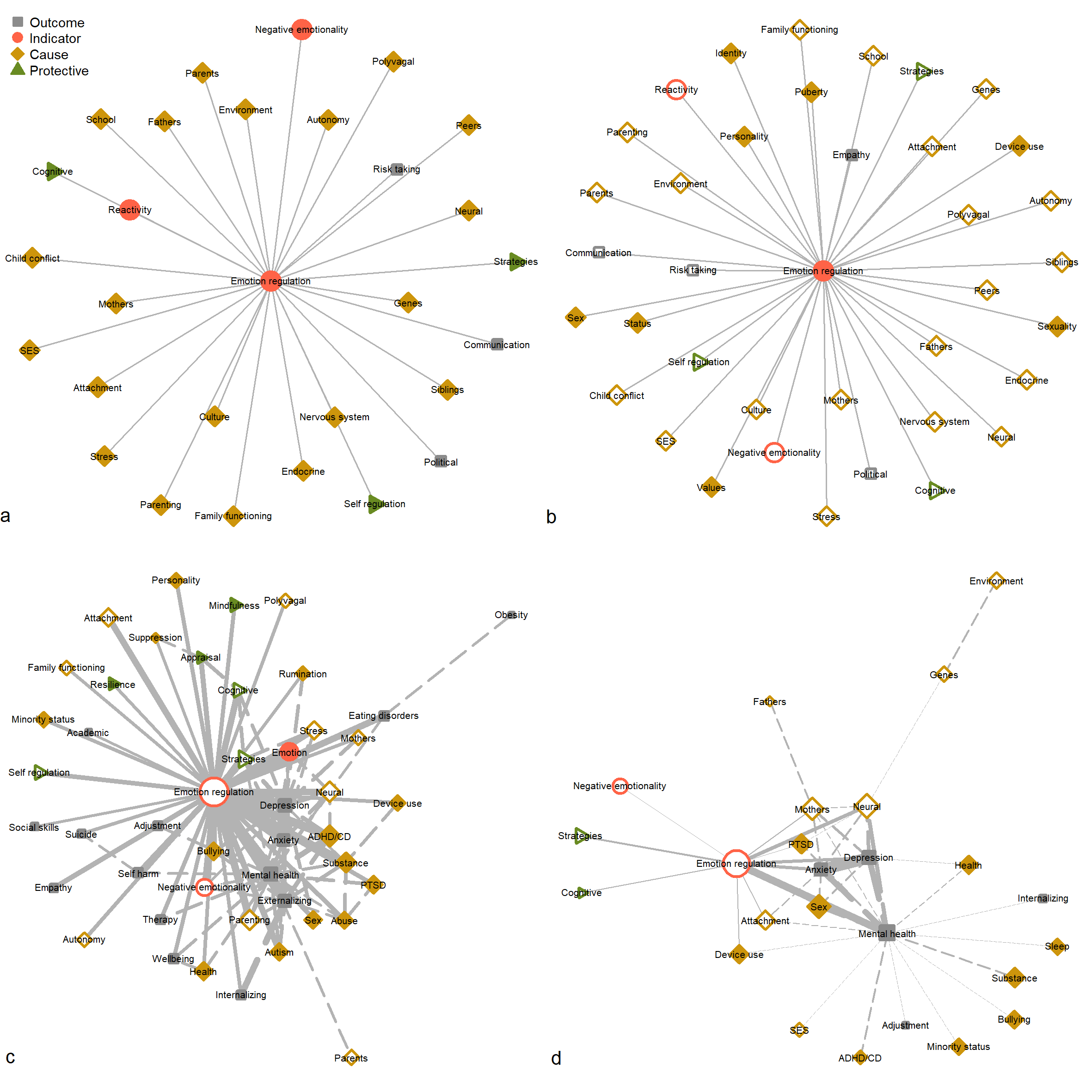

Supplement: Supplementary file 1 — Supplementary file1 (ZIP 54379 kb) [file 40894_2021_160_MOESM1_ESM.zip › veni_sysrev-master/networks.png]

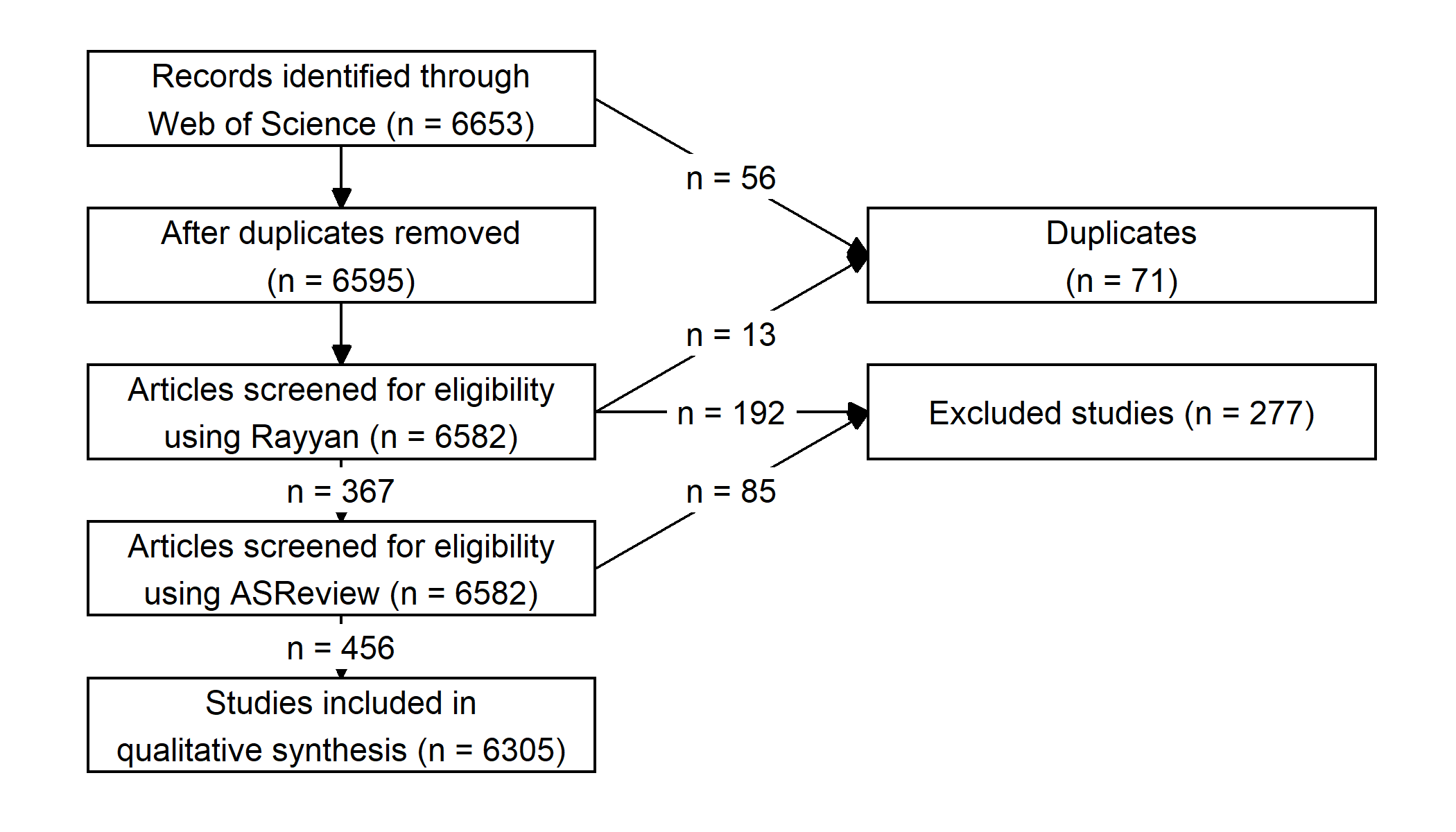

Supplement: Supplementary file 1 — Supplementary file1 (ZIP 54379 kb) [file 40894_2021_160_MOESM1_ESM.zip › veni_sysrev-master/prismachart.png]

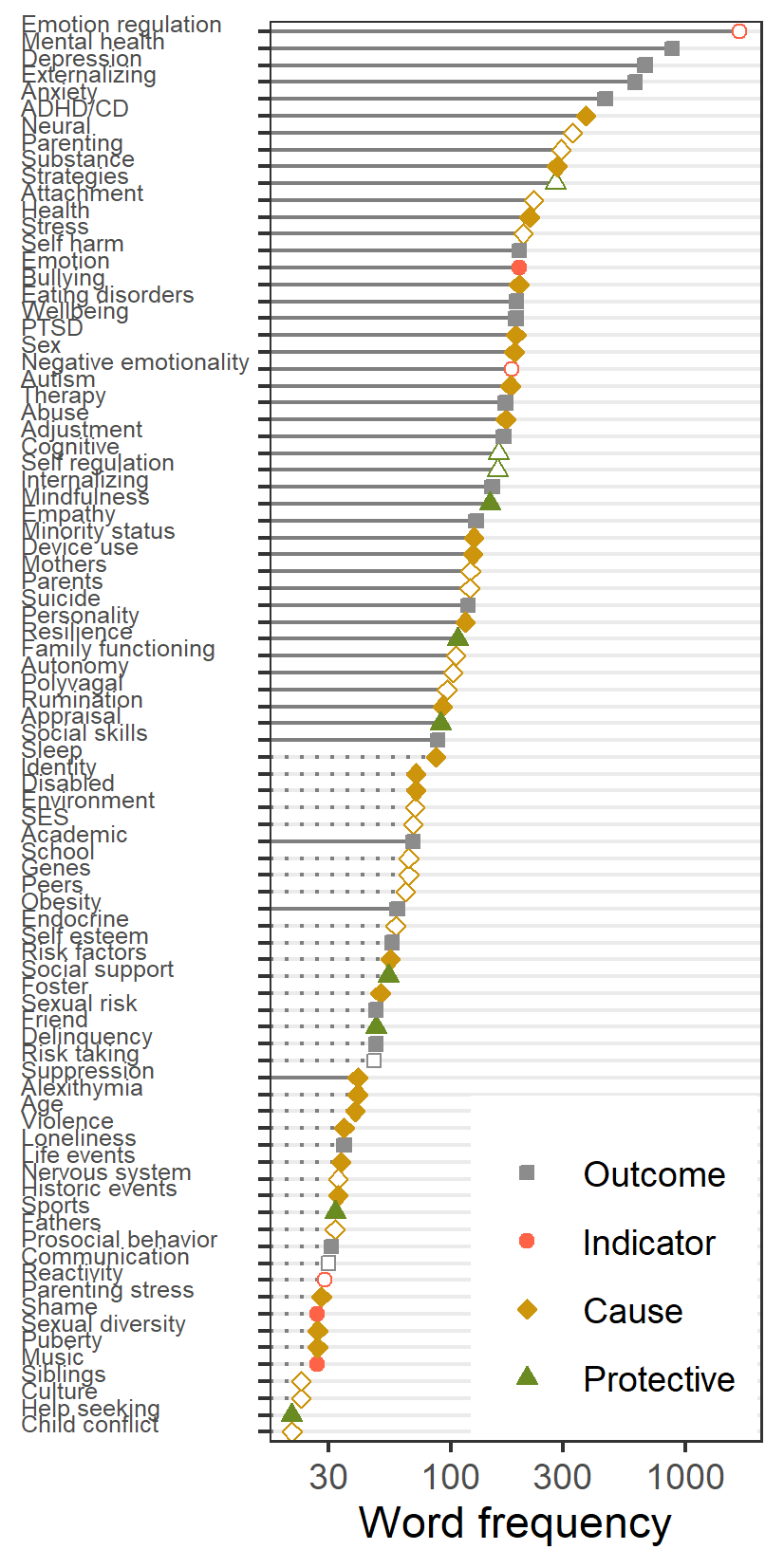

Supplement: Supplementary file 1 — Supplementary file1 (ZIP 54379 kb) [file 40894_2021_160_MOESM1_ESM.zip › veni_sysrev-master/s1_varimp.png]

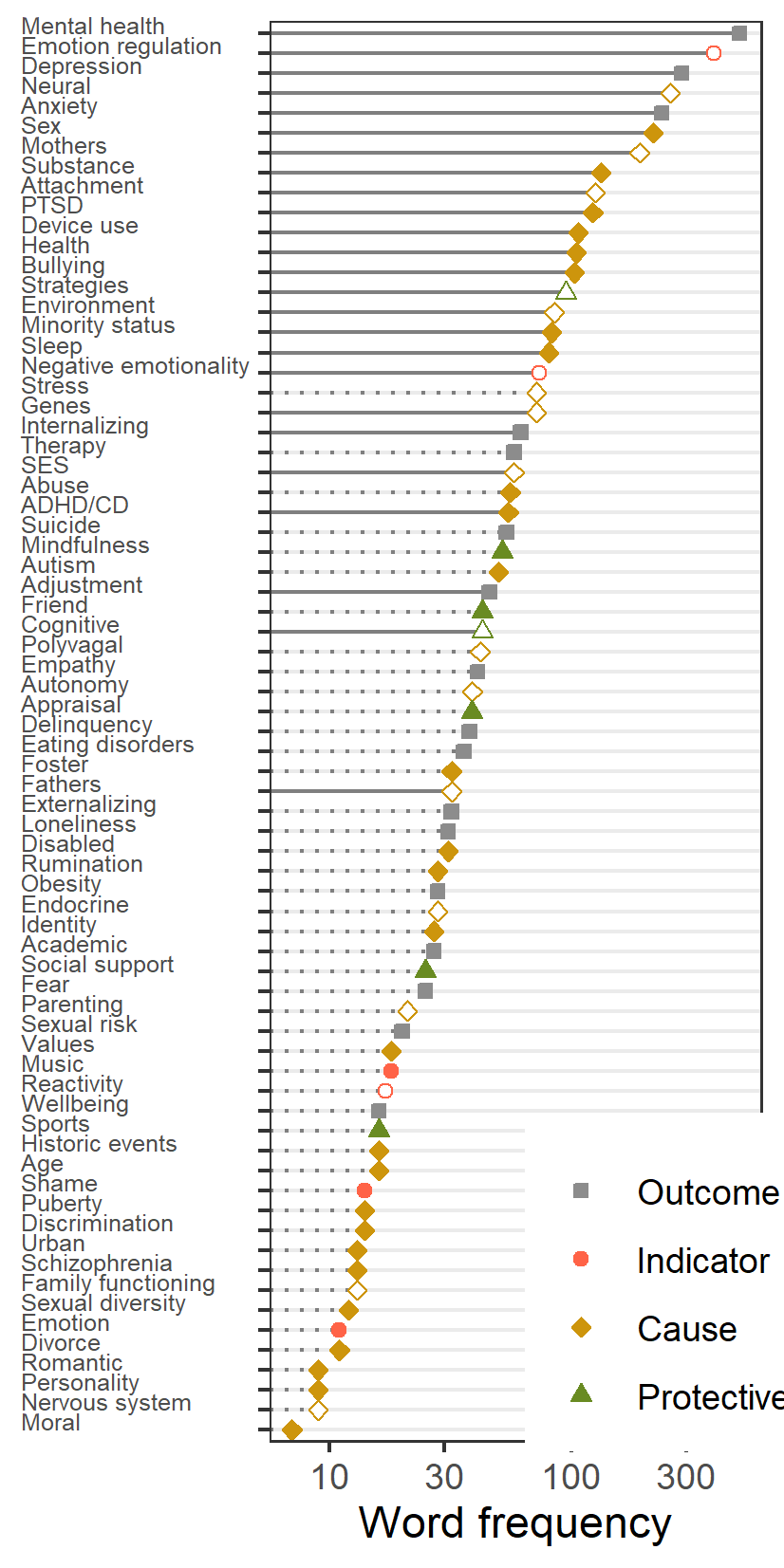

Supplement: Supplementary file 1 — Supplementary file1 (ZIP 54379 kb) [file 40894_2021_160_MOESM1_ESM.zip › veni_sysrev-master/s2_varimp.png]

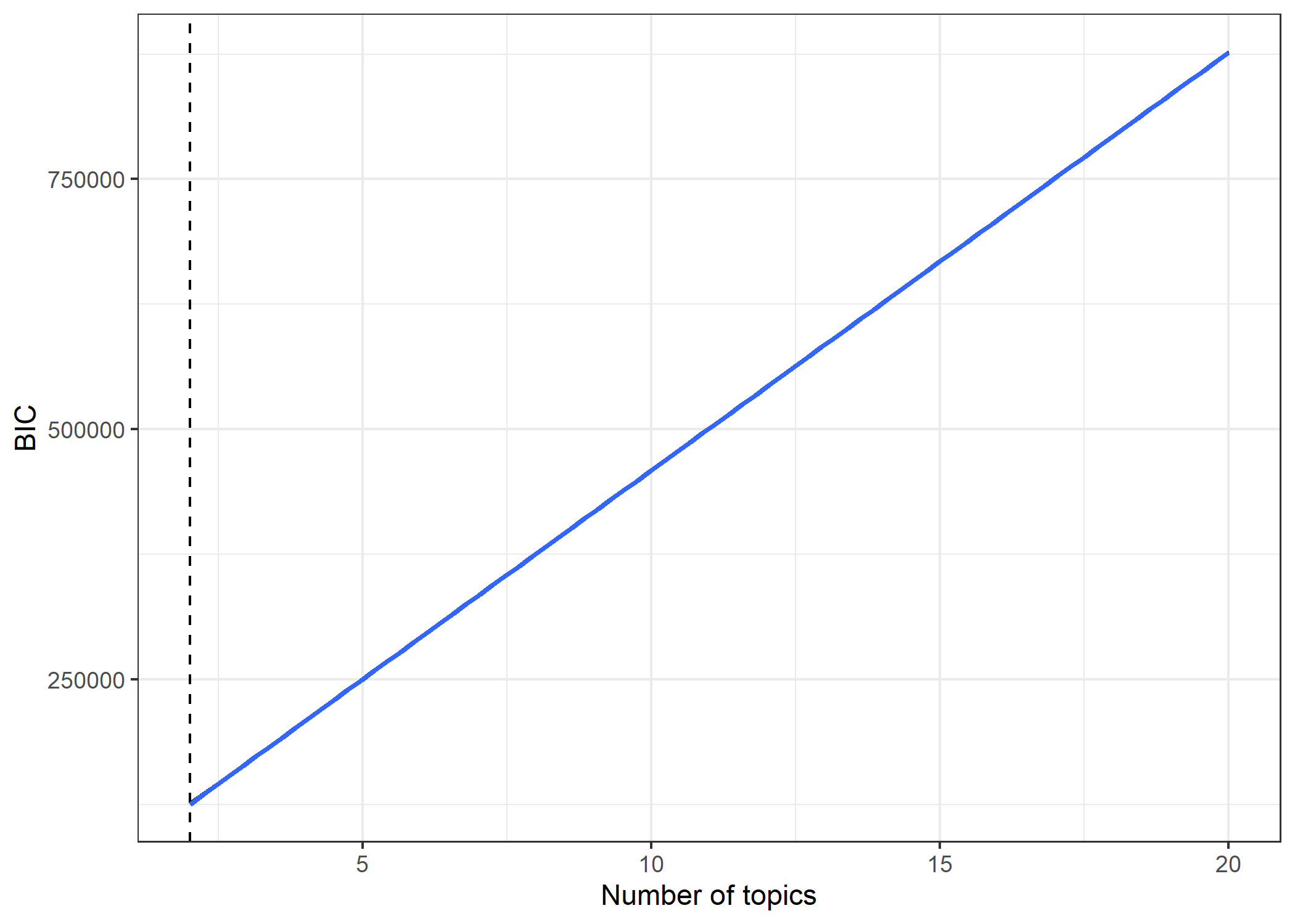

Supplement: Supplementary file 1 — Supplementary file1 (ZIP 54379 kb) [file 40894_2021_160_MOESM1_ESM.zip › veni_sysrev-master/study1_BIC.png]

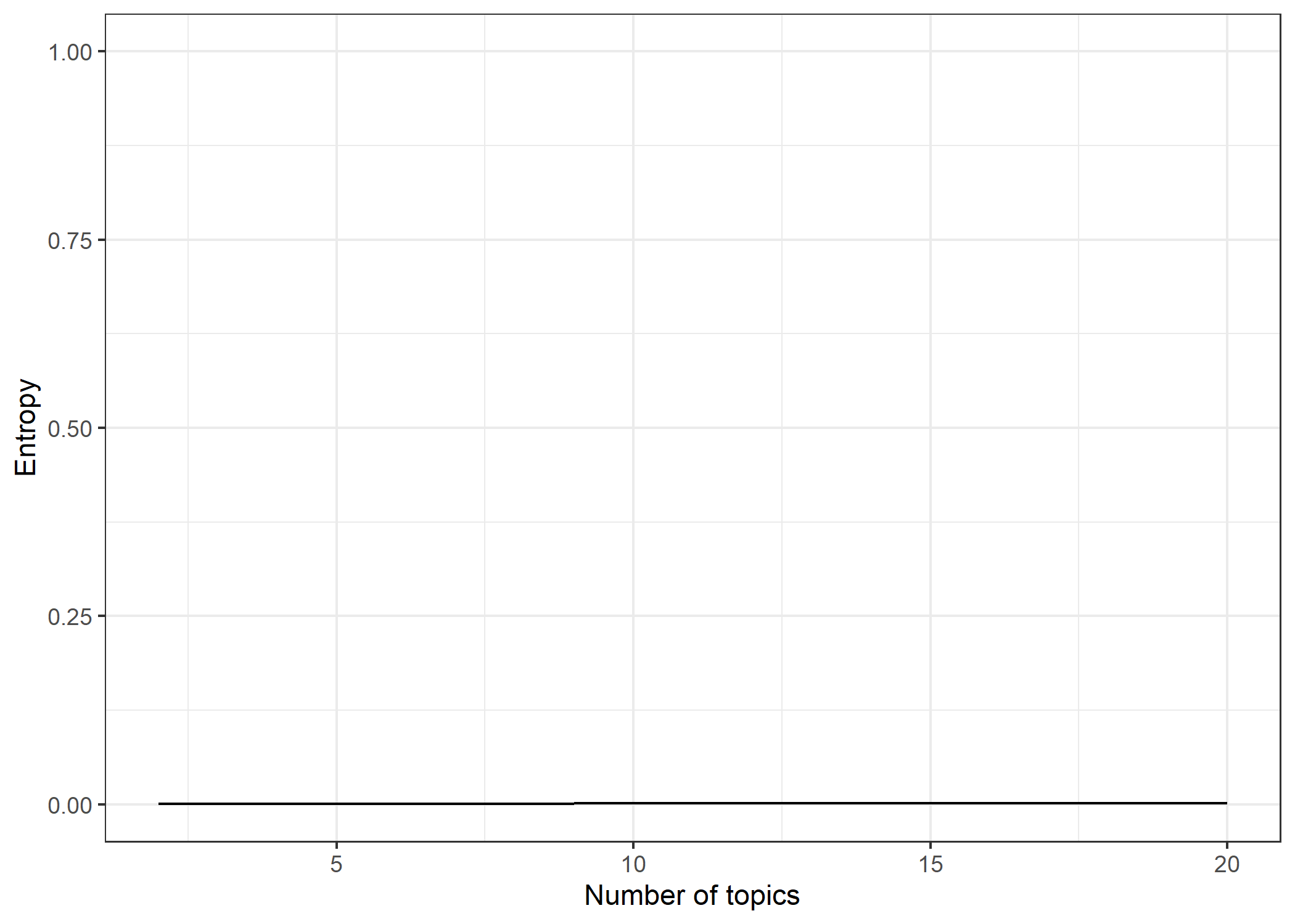

Supplement: Supplementary file 1 — Supplementary file1 (ZIP 54379 kb) [file 40894_2021_160_MOESM1_ESM.zip › veni_sysrev-master/study1_entropies.png]

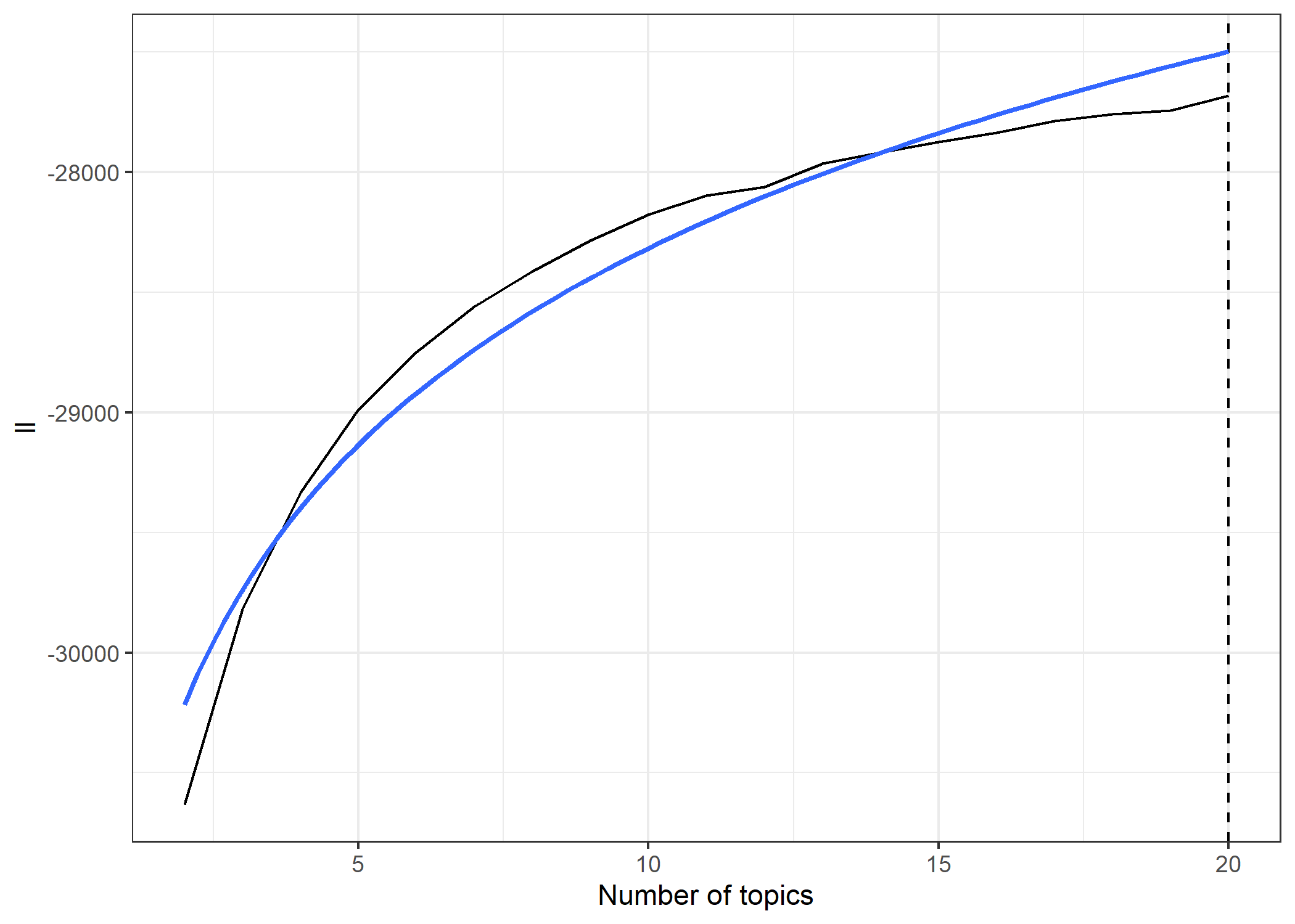

Supplement: Supplementary file 1 — Supplementary file1 (ZIP 54379 kb) [file 40894_2021_160_MOESM1_ESM.zip › veni_sysrev-master/study1_ll.png]

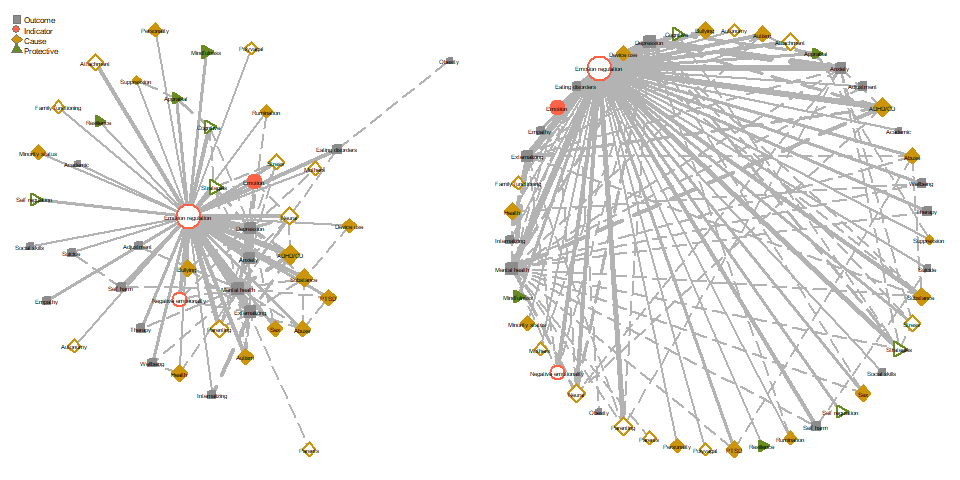

Supplement: Supplementary file 1 — Supplementary file1 (ZIP 54379 kb) [file 40894_2021_160_MOESM1_ESM.zip › veni_sysrev-master/study1_network1.png]

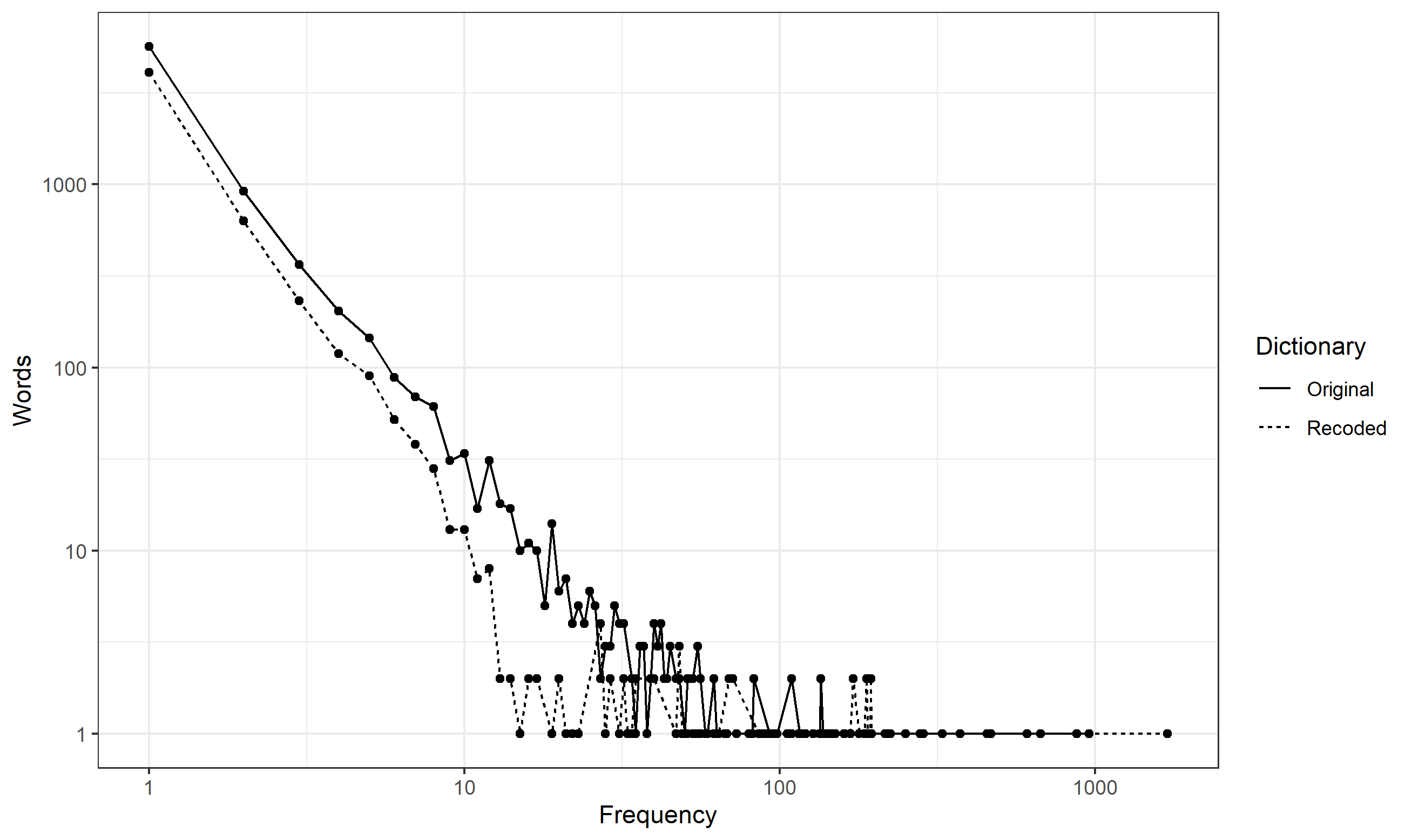

Supplement: Supplementary file 1 — Supplementary file1 (ZIP 54379 kb) [file 40894_2021_160_MOESM1_ESM.zip › veni_sysrev-master/study1_word_frequency.png]

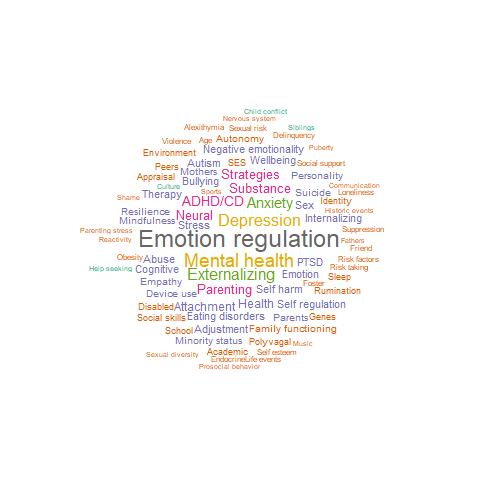

Supplement: Supplementary file 1 — Supplementary file1 (ZIP 54379 kb) [file 40894_2021_160_MOESM1_ESM.zip › veni_sysrev-master/study1_wordcloud.png]

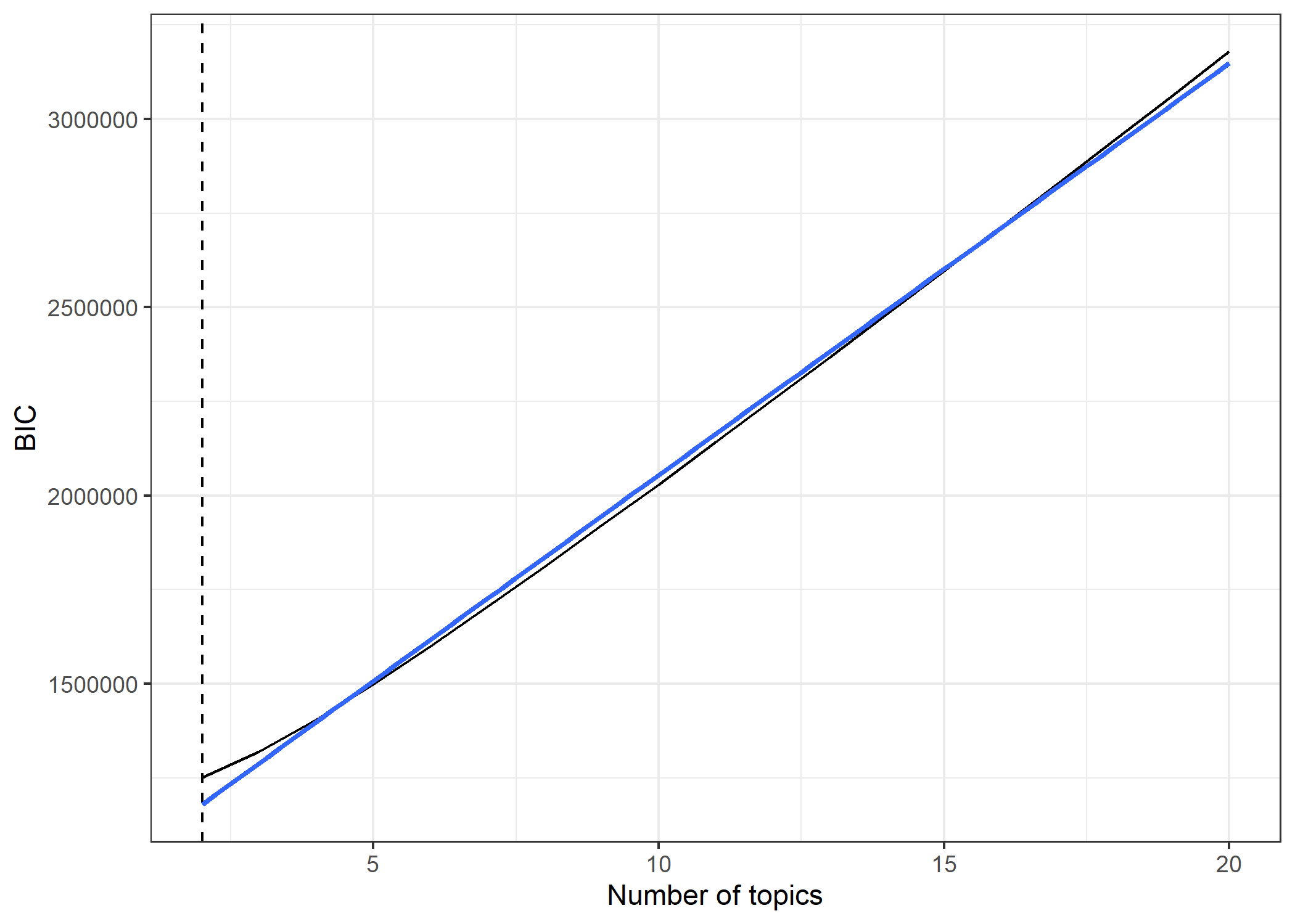

Supplement: Supplementary file 1 — Supplementary file1 (ZIP 54379 kb) [file 40894_2021_160_MOESM1_ESM.zip › veni_sysrev-master/study2_BIC.png]

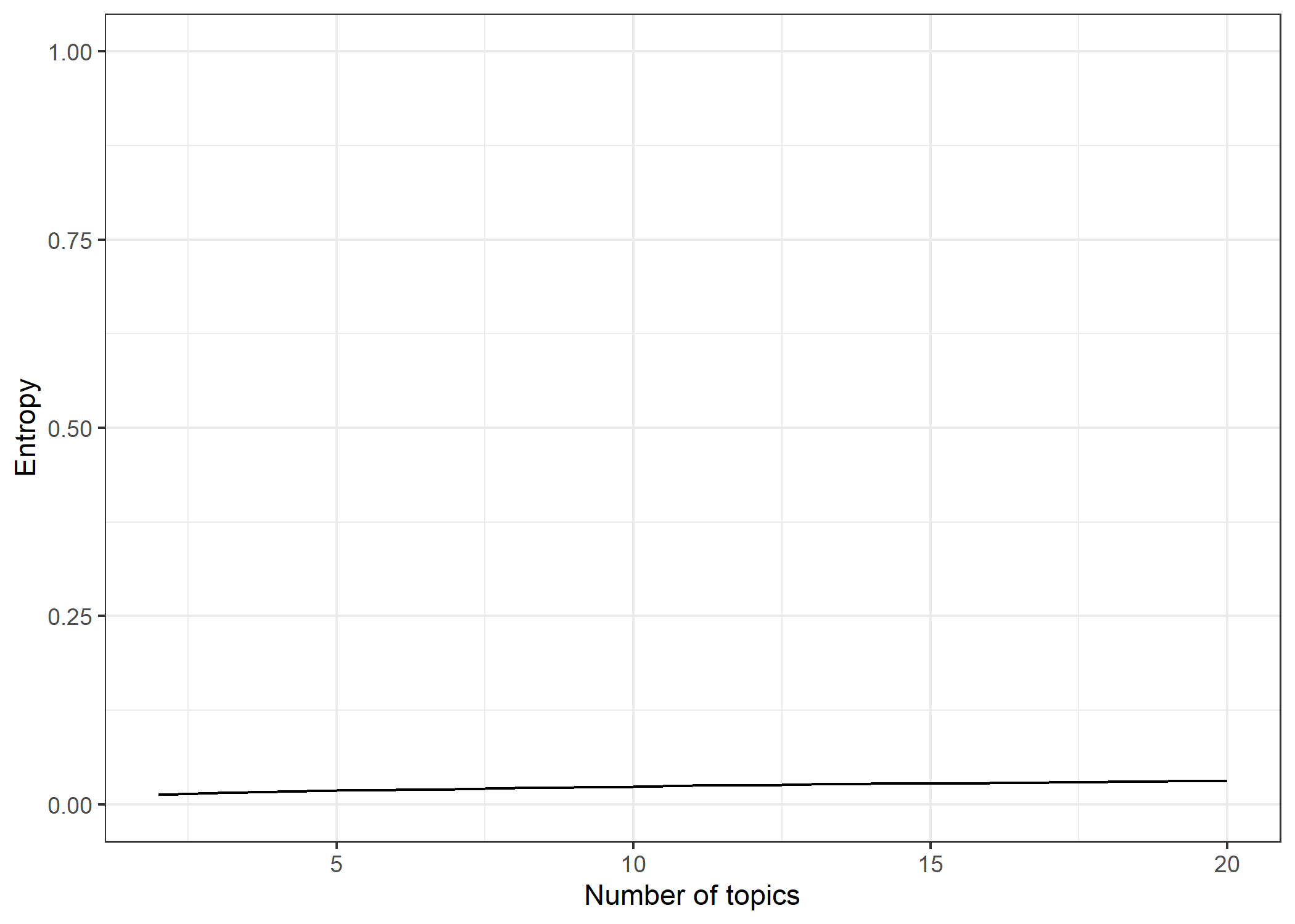

Supplement: Supplementary file 1 — Supplementary file1 (ZIP 54379 kb) [file 40894_2021_160_MOESM1_ESM.zip › veni_sysrev-master/study2_entropies.png]

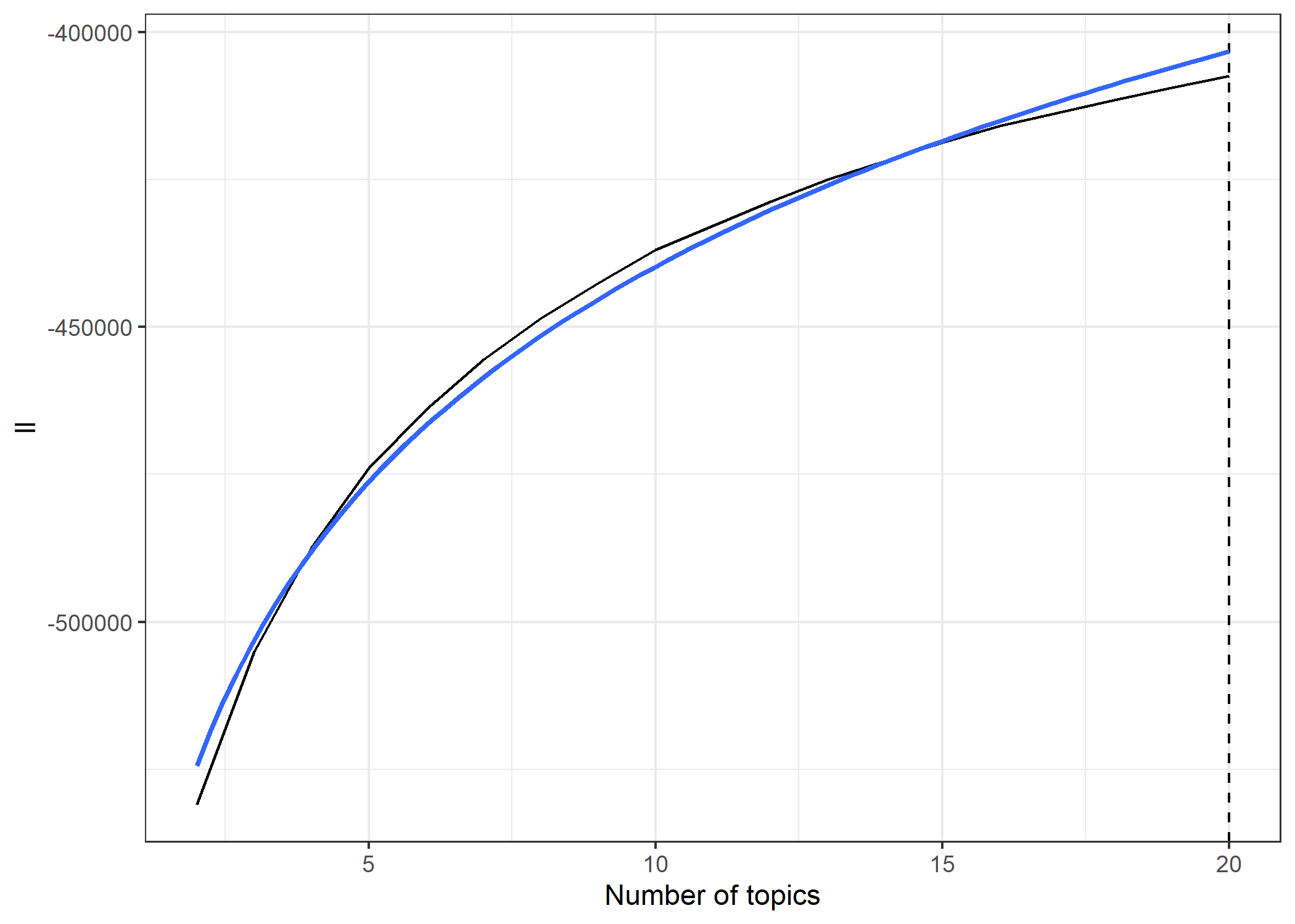

Supplement: Supplementary file 1 — Supplementary file1 (ZIP 54379 kb) [file 40894_2021_160_MOESM1_ESM.zip › veni_sysrev-master/study2_ll.png]

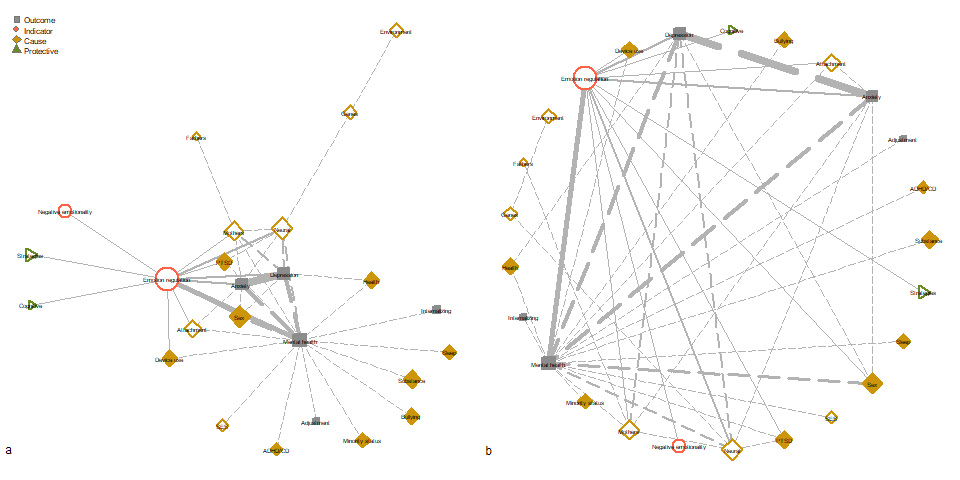

Supplement: Supplementary file 1 — Supplementary file1 (ZIP 54379 kb) [file 40894_2021_160_MOESM1_ESM.zip › veni_sysrev-master/study2_network1.png]

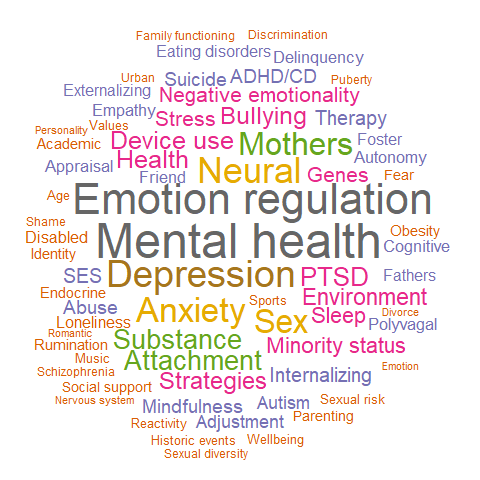

Supplement: Supplementary file 1 — Supplementary file1 (ZIP 54379 kb) [file 40894_2021_160_MOESM1_ESM.zip › veni_sysrev-master/study2_wordcloud.png]

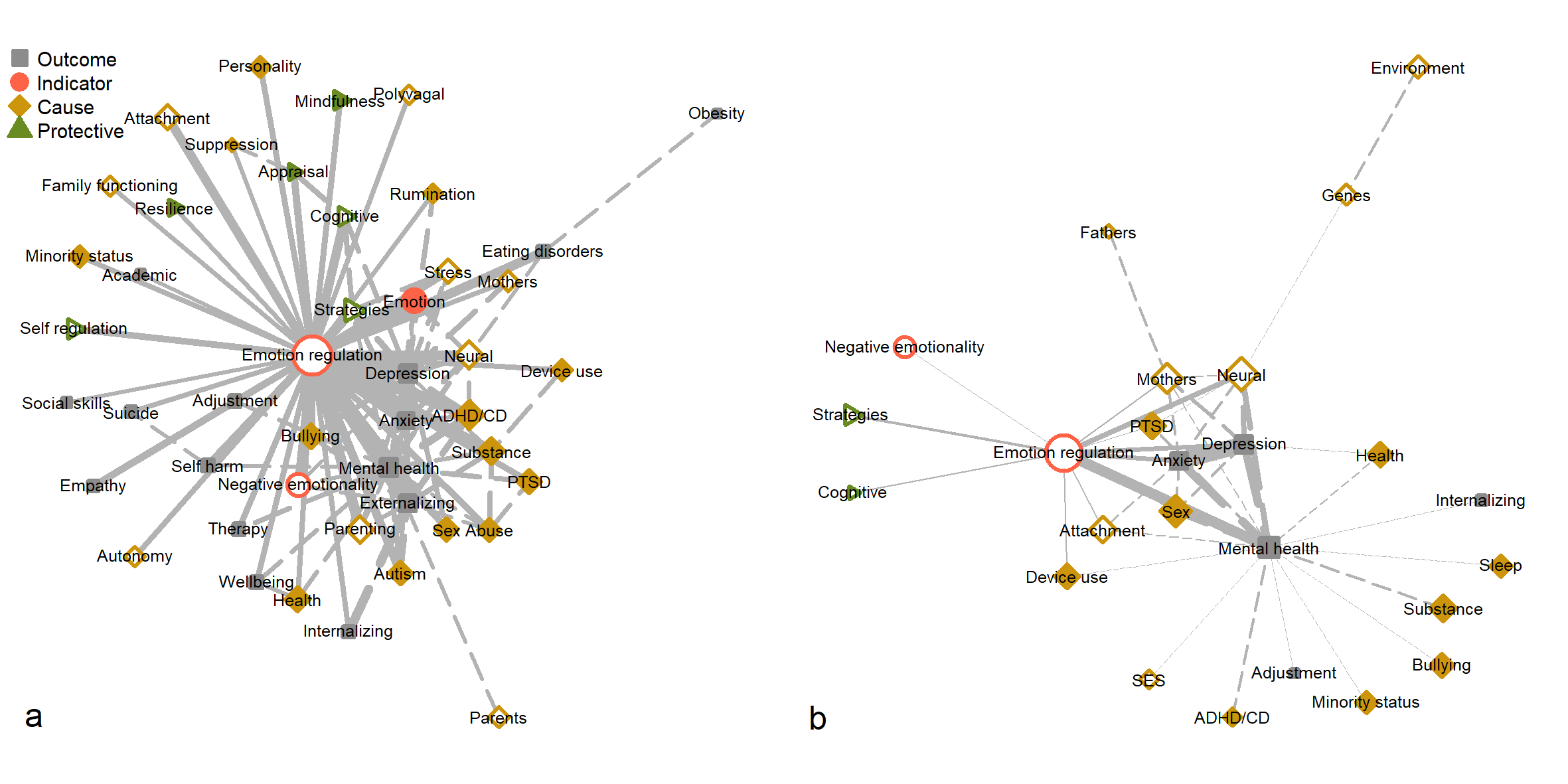

Supplement: Supplementary file 1 — Supplementary file1 (ZIP 54379 kb) [file 40894_2021_160_MOESM1_ESM.zip › veni_sysrev-master/tmnetworks.png]

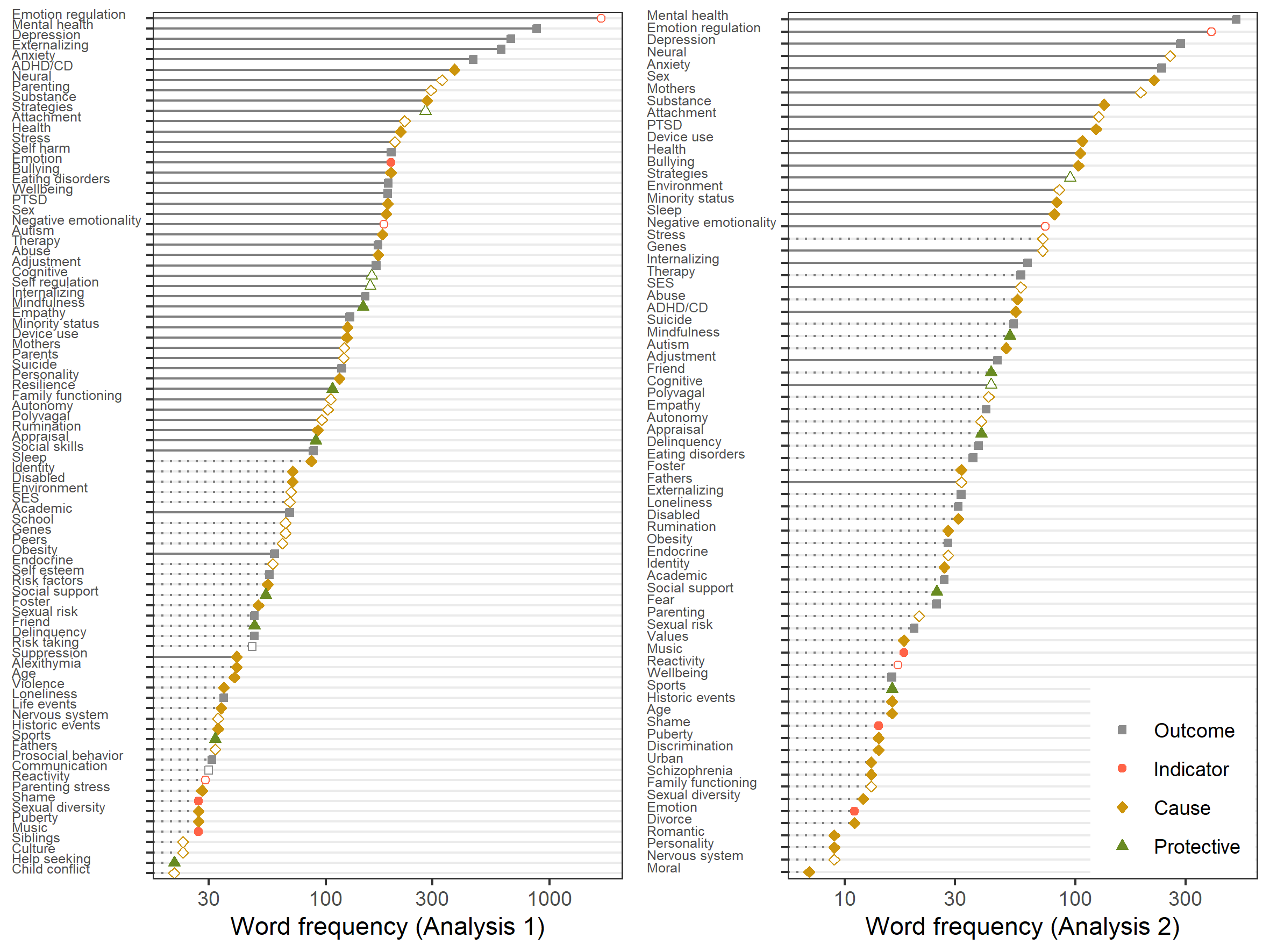

Supplement: Supplementary file 1 — Supplementary file1 (ZIP 54379 kb) [file 40894_2021_160_MOESM1_ESM.zip › veni_sysrev-master/varimps.png]

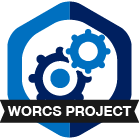

Supplement: Supplementary file 1 — Supplementary file1 (ZIP 54379 kb) [file 40894_2021_160_MOESM1_ESM.zip › veni_sysrev-master/worcs_badge.png]
