# Supplementary material for: Mapping Phenomena Relevant to Adolescent Emotion Regulation: A Text-Mining Systematic Review
Source: Adolesc Res Rev. 2021 May 21;7(1):127–39. doi: 10.1007/s40894-021-00160-7 (PMC8138847; doi:10.1007/s40894-021-00160-7)
Supplement: Supplementary file 1 — Supplementary file1 (ZIP 54379 kb) [file 40894_2021_160_MOESM1_ESM.zip › veni_sysrev-master/action_letter.html]

Action letter


# Action letter

#### Caspar J. van Lissa

#### 2/11/2021

# Reviewer #1:

**Reviewer 1, comment 1:** The abstract is lacking in two regards:

**Reviewer 1, comment 1a:** The actual findings are not reported

**Response:** As requested, we have summarized the findings in the abstract. The abstract now reads:

> Adolesence is a developmentally sensititve period for emotion regulation with potentially lifelong implications for mental health and well-being. Although substantial empirical research has addressed this topic, the literature is fragmented across subdisciplines, and an overarching theoretical framework is lacking. The first step towards constructing a unifying framework is identifying relevant phenomena. This systematic review of 6305 papers used text mining to identify phenomena relevant to adolescents’ emotion regulation. We first established a baseline of relevant phenomena discussed in theory and recent narrative reviews. We then conducted text mining analyses of article keywords and abstracts, examining term frequency as an indicator of relevance and term co-occurrence as an indicator of association. Results reflected themes commonly featured in theory and narrative reviews, such as socialization and neurocognitive development, but also identified undertheorized themes, such as developmental disorders, physical health, external stressors, structural disadvantage, substance use, identity and moral development, and sexual development. This illustrates how text mining systematic reviews (TMSR), a novel approach, may complement narrative reviews. Future theoretical work might seek to integrate these undertheorized themes into an overarching framework, and empirical research might consider them as promising areas for future research, or potential confounders in research on adolescents’ emotion regulation.

**Reviewer 1, comment 1b:** it is not clear how the findings integrate te literature.

**Response:** Perhaps this turn of phrase was somewhat unfortunate. When discussing the relevance of the study in the abstract, we now simply state:

> Future theoretical work might seek to integrate these undertheorized themes into an overarching framework, and empirical research might consider them as promising areas for future research, or potential confounders in research on adolescents’ emotion regulation.

**Reviewer 1, comment 2:** Key words are not provided.

**Response:** We apologize for the oversight; the keywords were not retained when converting the manuscript to the required format for ADRR. We have reinstated them.

**Reviewer 1, comment 3:** The title is about dysregulation but the first paragraph is all about regulation. The manuscript would be more effective if it were more consistent.

**Response:** We agree that greater consistency would improve the clarity of the writing, and have changed the manuscript to use the more general term “emotion regulation” throughout.

**Reviewer 1, comment 4:** The first paragraph [promises] that the results will help unify theory and address limitations of research areas that the author deems in silos. This is a heavy burden to carry.

**Response:** It was not our intention to give the impression that “our results will help unify theory and address limitations of research areas that the author deams in silos”.

First of all, the Reviewer credits us for the observations that research areas are “in silos”, but this is a commonly mentioned limitation of the existing literature. To address this issue, and clarify that this is not our original claim, we have included additional references. The revision now reads:

> Despite the abundance of publications in this field, several prior theoretical and empirical reviews have argued that the literature is somewhat fragmented, because different (sub)disciplines have approached the issue in disparate ways, in the absence of consistent terminology and unifying theory (Buss, Cole, and Zhou 2019; Bariola, Gullone, and Hughes 2011; Stifter and Augustine 2019).

Second of all, the Reviewer’s comments indicate that the intended contribution of the present study was insufficiently clear. Our results will not unify theory, but it does lay the foundation for future work that intends to do so, as identification of the relevant empirical phenomena is the first step in theory construction methodology. In the revision, we have attempted to clarify the intended contribution of the present study as follows:

> This has led to recent calls for the consolidation of empirical insights into overarching theory (e.g., Buss, Cole, and Zhou 2019). Formal methods for theory construction state that the first step in this endeavor is to identify the relevant *empirical phenomena* (**???**; **???**), defined as stable and general features of the world (**???**). We propose that systematic reviews are a suitable method for taking this first step, based on the assumption that phenomena considered to be stable and general will be more frequently represented in the published literature. Traditional narrative reviews have some limitations, however, as they been shown to be marred by small convenience samples, confirmation bias, and an undue emphasis on positive results (Littell 2008). We intend to overcome these limitations by adopting a novel method: the text mining systematic review (TMSR).

Third of all, the Reviewer’s comment indicates that we have raised expectations that we would “address limitations of research areas that the author deams in silos”. It is not the case. A systematic review like the present paper is meta-research. Meta-research describes bodies of primary research. It therefore cannot address limitations at the level of primary research. The only limitation that we address is at the meta-research level: The lack of a comprehensive overview of factors relevant to adolescent emotion regulation in prior theory and reviews. We have attempted to address this comment by clarifying the intended contribution of the study throughout the manuscript.

**Reviewer 1, comment 5:** The entire section on page 3: it is not clear why this is presented. It could be presented in a brief sentence and likely should because it does not really relate to what is reviewed. If it is kept, it should be edited so it relates to the actual review. The paragraph on page 4 may be relevant, but it is not clear how it eventually relates to the study.

**Response:** These two paragraphs were intended to substantiate the relevance and scope of the present study. Based on the Reviewer’s comment, we now see that this was perhaps redundant with the opening paragraphs, which already provided rudimentary justification of the relevance and scope. We have thus attempted to address the Reviewer’s comment by cutting these paragraphs on pages 3 and 4, merging the core arguments with the opening paragraphs.

The opening paragraphs now read:

> Adolescence is a life stage ranging from pubertal onset to adult-like independence (Steinberg 2014; Sawyer et al. 2018). A key developmental challenge in this stage is acquiring mature emotion regulation skills (Crone and Dahl 2012; Zimmermann and Iwanski 2014). Adolescents experience biological, cognitive, and social changes that prompt new emotional experiences and tax regulatory abilities (Steinberg 2014). These coalescing changes temporarily restrict adolescents’ capacity for emotion regulation (Zimmermann and Iwanski 2014), resulting in more frequent and intense (negative) emotions (see Silk, Steinberg, and Morris 2003). Although most adolescents eventually develop mature emotion regulation abilities, for as many as one in five (Lee et al. 2014), adolescence marks the onset of life course persistent emotion regulation-related psychopathology (see also Aldao, Nolen-Hoeksema, and Schweizer 2010; Schäfer et al. 2017). Given the prevalence of emotion regulation difficulties in adolescence, their implications for mental health and downstream cost to society, it is important to have a comprehensive overview of the factors associated with adolescents’ emotion regulation.

**Reviewer 1, comment 6:** The existing theoretical landscape is presented in a way that is unclear how it really relates to the review.

**Response:** We understand that it is essential to clarify why we present this brief theoretical review before diving into the results of the present study. In order to address this comment, we now explicitly state why a review of the existing theoretical landscape is relevant to the present systematic review. The revised manuscript reads:

> Although it has been argued that there is a paucity of theories *specific to* adolescent emotion regulation, many *relevant* theories are commonly invoked in empirical work (see Buss, Cole, and Zhou 2019). In order to contextualize the results of our systematic review and assess to what extent the identified phenomena through text mining mirror or complement existing theory, we provide a brief review of the existing theoretical landscape.

**Reviewer 1, comment 7:** Figure 1 on page 5: it is not clear why it is there. It should come after the introduction. And, in fact, it should appear after the references and the text should simply have a placeholder for the Figure. It should be referred to at the end of the introduction (before the current study section).

**Response:** As requested by the Reviewer, we have placed the Figure after the References section, retaining only a placeholder in-text. As requested, we also refer to the Figure at the end of the Introduction:

> There are notable parallels between phenomena relevant to adolescents’ emotion regulation as identified in these narrative reviews, and in the preceding theoretical literature, as can be seen in Figure 1.

**Reviewer 1, comment 8:** The figure nicely points to outcomes, indicators, causes and protective factors. That is not clear from the introductory materials.

**Response:** In the previous manuscript, we had merged the two Figures from the theoretical Introduction with the two Figures from the Results section in order to facilitate comparisons between the two. We see now that this caused some confusion. In order to address this comment, we have separated the Figures from these two sections, and we have added the following information about the classification of phenomena:

> In reading the theoretical literature, we documented relevant phenomena and whether these were discussed as putative causes, outcomes, indicators, or protective factors in relation to emotion regulation. The left panel of Figure @ref(fig:figbaseline) visually summarizes the phenomena discussed in the theoretical literature, using a style similar to our subsequent systematic review, in order to facilitate contextualizing our original findings.

**Reviewer 1, comment 9:** The section on theory is not presented in a way that would have it appear as authoritative. It reads more like a hodgpode of theories.

**Response:** The Reviewer’s point is well-taken, and yet one of the main motivations for the present study is the fact that the theoretical landscape *is indeed a bit of a hodgepodge*. This has been stressed by several prior reviewers, as pointed out in our response to the Reviewer’s 4th comment. We feel that any attempt to impose an apparently “authoritative” structure on this messy field would be somewhat misleading, and would undermine the intended contribution of the present study. To address the Reviewer’s comment, we have substantially edited the theoretical review for increased readability, while explicitly acknowledging the lack of overarching structure.

**Reviewer 1, comment 10:** The section would be stronger if it also gave readers a sense of which theories are deemed important/influential and worth using. There needs to be a sense that the important theories are being examined.

**Response:** We are happy to do so if there is an objective way to do so, but it’s not clear what metric should be used. Citation counts are obvious but problematic - they are confounded with publication date (favoring Bronfenbrenner and Sameroff), some theories are influential but rarely credited (e.g., polyvagal theory), and other theories are published by different authors at approximately the same time (e.g., the maturity gap was described approximately simultaneously by Casey, by Crone, and by Cracco). We are open to other suggestions.

Importantly, as we have argued in the paper, the state of theory in the field is a bit of a “hodgepodge”, and much research lacks explicit theoretical foundations. The theories that *are* available are limited because they are not uniquely suited to this particular area of research, and some are vague but all-encompassing macro-theories (e.g., Bronfenbrenner, Sameroff), and others are concrete and specific, but not embedded in an overarching framework (e.g., Crone, Morris). Pending further guidance on how to address this comment, we have made an effort to reinforce these points throughout the manuscript.

**Reviewer 1, comment 11:** The first paragraph on page 9 needs to be edited. It appears too haphazard. (Why use “Finally,” as uo were not enumerating.) The problem, just as with the literature review, is that the studies appear presented randomly. One study did this, one did that, and so forth.

**Response:** We have rewritten this as requested. We have attempted to clarify that we are not presenting many different studies, but merely summarizing one seminal literature review, and one recent “update” that covers more recent literature in greater detail. We have further organized the phenomena discussed in these two reviews in line with the classification of Bronfenbrenner and Sameroff, introduced in the opening paragraph on relevant theory. We thus begin with phenomena at the individual level, followed by proximal influences (microsystem), followed by distal influences (mesosystem and exosystem).

**Reviewer 1, comment 12:** The fundamental problem with the “The present paper” section is that it really does not do what is needed. It is a plan of analysis. This section should note the research questions and hypotheses.

**Response:** We agree with the Reviewer that “The present paper” section was basically written as a plan of analysis.

To address this comment, we have rewritten this section to be more in line with the APA guidelines, by summarizing the study design, linking it to the study aims, and stating the intended outcomes. Note that we cannot honor the Reviewer’s request for hypotheses, because this is an inductive (exploratory) study, and as such, it does not test any hypotheses. To avoid confusion about this point, we now emphasize the inductive nature of the study throughout the manuscript, including in “The present paper”. This section now reads:

> The present paper set out to map the phenomena relevant to adolescent emotion regulation using a text mining systematic review (TMSR) of the literature. This approach considers the frequency with which a phenomenon is covered in the literature to be indicative of its relevance, and the frequency with which phenomena are investigated together within publications to be indicative of a relationship between these constructs. The expected outcomes consist of term frequency metrics, which can be rank ordered to identify the most relevant phenomena, and term co-occurrence metrics. These metrics can be jointly visualized as a network graph, thereby “mapping” phenomena relevant to adolescent emotion regulation. Note that this study is inductive (exploratory), as opposed to deductive (confirmatory) research, and as such does not test any hypotheses (see De Groot’s work on the empirical cycle **???**)(footnote: Note that aside from induction, the term “abduction” is also used in the methodological literature. We consider injunction to refer to inferences based purely on observations (data). Abduction differs from induction because it additionally involves the attribution of observations to explanatory principles).

**Reviewer 1, comment 13:** By the time I get to eh methods section, including the beginning of this section, I still see a focus on emotion regulation, not emotion dysregulation that appears in the title.

**Response:** We have addressed this comment as requested; see the response to R1C3.

**Reviewer 1, comment 14:** As I read the materials on pages 11 and 12, I see a heavy reliance on the computerized analytical system to do the research and coding. I think it would be helpful to know what decisions are made by the authors and, if there are any, we should know about reliability checks and, if there are none, that still should be noted.

**Response:** This is a crucial comment with far-reaching implications. The Reviewer asks what decisions are made by the authors. The straightforward answer is: all decisions. The reason for this is that inductive / exploratory research affords considerable creative license to the researcher (**???**; **???**). This is because the number of ways to conduct exploratory analysis on a single data set are infinite. The present paper represents just one possible way. The “computerized analytical system”, as referred to by the Reviewer, is an explicit representation of our subjective decisions in analyzing the data in the form of code.

The Reviewer further asks for reliability checks, also known as a sensitivity analysis or multiverse analysis (**???**). Such reliability checks are only defined, however, in the context of deductive (confirmatory) analysis, where they are used to ensure that hypothesis tests / statistical inferences are robust to permutations of the data processing strategy. Reliability checks are undefined in the context of the present study, because it is inductive (exploratory), no hypotheses are tested nor inferences are drawn, and the number of alternative possible analyses is infinite.

We do emphatically agree with the Reviewer that it is important to be clear about what decisions are made by us.

To address this comment, we have taken the following steps:

1. We have documented the methods in as much detail as possible in the manuscript, and added new online supplemental analyses for more complex analyses that we could not describe in sufficient detail.
2. We have made all analysis code and data publicly available in an open access repository. This repository also contains the entire historical development of this study, so readers can see that our analysis proceeded in a straightforward way. We mention this explicitly in the revised paper.
3. Throughout the manuscript, including in the revised Methods section, we now emphasize that these are inductive (exploratory) analyses, and as such, depend on subjective decisions:

> As the purpose of inductive research is to inform hypothesis generation, this approach affords the researcher with substantial creativity (**???**). This means that subjective decisions are made throughout the analysis process. To ensure that all such decisions are properly documented, we have made all code, data, and the historical record of this project publicly available in an online research repository at [masked]. We used the Workflow for Open Reproducible Code in Science (WORCS, **???**) to make the analyses reproducible. We encourage reuse of the analysis code, and secondary analysis of the data.

4. The revised Limitations section now addresses this issue:

> One final limitation is that several subjective decisions were made throughout the analysis process. This limitation is inherent to inductive studies, however (e.g., **???**): There are infinite possible ways to conduct exploratory analysis, and researchers have considerable creative license in doing so. We have attempted to address this limitation by explicitly mentioning important decisions in the manuscript where possible, and by making all analysis code and data publicly available in a fully reproducible format (based on **???**), so that others may explore alternative exploratory analysis strategies.

**Reviewer 1, comment 15:** At the bottom of page 12, it is clear that a decision is made in terms of retaining within a percentile. How do we know if that is the right decision? There appear to be decisions like this made throughout. Given the reliance on the program, we need to know more.

**Response:** This is indeed a good example of a subjective decision. In our response to the previous comment, we explain that we now explicitly acknowledge each such subjective decision in the revised manuscript. In addition to the blanket statement we have added to the Methods section, we now also acknowledge the subjectivity of each decision like this one explicitly in the revised manuscript.

With regard to the Reviewer’s question - “How do we know if that is the right decision?” - there is no straightforward answer. We had thoroughly scoured the literature for established practice for pruning terms in text mining when preparing the initial submission, and did so again during revision, but most published analyses appear to either retain a fixed number of terms, or drop terms that occur less than a fixed number of times. Our selection method has the advantage of taking the distribution of word frequencies into account. It is thus more flexible than a fixed cutoff, and still successful in pruning terms that occur only a few times in the corpus. To address this comment, we now mention the fact that this is a subjective decision explicitly in the paper (see excerpt below). We also provide more quantitative information about the thresholds and the cut terms.

> This is a subjective criterion, but compared to the common practices of either retaining a fixed number of terms or pruning terms below a fixed frequency (see **???**), it has the advantage of being responsive to the empirical distribution of term frequencies. Note that the vast majority of pruned terms occurred only once in the corpus.

**Reviewer 1, comment 16:** Page 13, there is again pruning. Justifications for the cutoff should be noted. There is a manual classification and we are left with no sense of its reliability/validity.

Please see our response to the previous comment. Additionally, note that the majority of the pruned terms occurred in only one document, as can be seen in the plot below. Even if the pruning criterion is subjective, it is hard to argue that we should interpret every word that occurred in the corpus. Thus, we would argue, some pruning is necessary - and if a better method is available, we would be happy to use that instead.

```
df_plot <- cbind(as.data.frame(read_yaml("study1_term_freq_dist.yml")), Analysis = "Analysis 1")
names(df_plot)[1] <- "topterms"
df_plot <- rbind(df_plot, cbind(as.data.frame(read_yaml("study2_term_freq_dist.yml")), Analysis = "Analysis 2"))
df_plot <- df_plot[!df_plot$pruned, ]
df_plot$topterms <- ordered(df_plot$topterms, levels = sort(as.numeric(unique(df_plot$topterms))))
ggplot(df_plot, aes(x = topterms, y = Freq)) + geom_bar(stat = "identity") + theme_bw() + labs(x = "Frequency of term", y = "Number of terms") + facet_wrap(~Analysis, scales = "free")
```

**Reviewer 1, comment 17:** I really think the presentation would be more effective if it did not have results/discussions and then more results/discussions, and then a general discussion. We are not really dealing with different studies.

**Response:** As requested, we have rewritten these sections as a single Results and Discussion section. As this change spans several pages, we do not cite the entire revised section here, but refer to the revised manuscript.

**Reviewer 1, comment 18:** Page 20, the first full paragraph is a good example of text that is meant to show a contribution when it actually does not. Being the first or one of the first is not a contribution. What is more important is the actual finding. I realize that the focus here is on the method. But, being the first to use a method is not a contribution. What is important to highlight is the findings that come from the method, from the study.

**Response:** Point well taken; we have rewritten the paragraph in line with the Reviewer’s suggestions. We do still mention that we are the first to do this - but merely as a statement of fact, not as a contribution. The contributions section now reads:

> One strength of the present study is that it was more comprehensive than previous reviews of the literature in two respects (cf. Bariola, Gullone, and Hughes 2011; Coe-Odess, Narr, and Allen 2019): We conducted a systematic literature search, and we used text mining to comprehensively map the phenomena relevant to adolescents’ emotion regulation.  Using text mining offers unique advantages compared to narrative reviews (Littell 2008), because it can cover vastly greater corpora than a sentient reader, and follows a somewhat more objective, transparent, and reproducible procedure. As such, text mining systematic reviews offer an interesting perspective that complements theory and narrative reviews. Moreover, text mining systematic reviews offer an inductive approach to identify relevant phenomena in a particular area of research. This can serve as the first step towards formal theory construction (**???**). Our study was the first to implement this novel approach.

**Reviewer 1, comment 19:** Page 21, the political push for open science is not really a point that is relevant. If the authors wanted to review the actual papers, they could. They just need to identify the papers, download them, and search. Right? At any rate, I would avoid making political statements that, essentially, make the point that others should make their data available so that authors who use this method could use their method. Much of what is said here is not really reading well.

**Response:** This is an important comment, which we will address in four main points.

First, the Reviewer writes that “the political push for open science is not really a point that is relevant.” We agree with this sentiment. Our writing was not intended as such, and we have rewritten the offending section to avoid misunderstanding. In particular, we have removed the more general term “open science”, as we are really speaking only of open access publishing.

Second, although the *political* implications of open access publishing are not relevant here, its *methodological* implications are. In order to conduct meta-scientific research of any kind - be it systematic reviews, meta-analyses, or indeed text-mining - publications must be accessible. Open access publishing thus has methodological implications for meta-scientific research, and as such, is well within scope of the Limitations section of the present paper.

Third, the Reviewer writes “If the authors wanted to review the actual papers, they could [just] identify the papers, download them, and search. Right?”. Unfortunately, the approach suggested by the Reviewer is not feasible, for two reasons.

- The proposed approach is not scalable: Assuming that it would take only 10 minutes to find, download, and archive a full-text publication, it would have taken 131.35 working days to assemble the corpus for the present study (6305 papers \* 10 minutes)/(60 minutes \* 8 hours per day). To manually curate a larger dataset, such as CORD-19 (publications about the coronavirus), would take 8333.33 days (400.000+ papers \* 10 minutes)/(60 minutes \* 8 hours per day). As we suggest, an API - a programmatic interface for downloading full-text publications - would overcome this bottleneck.
- It is important to consider that not every scholar has the privilege to simply download papers. Aside from the *cost* of the aforementioned man-hours (which could be addressed by the API we propose), not everyone has comprehensive access to paywalled publications. Thus, such research could only be conducted at the world’s most well-funded universities.

Finally, the Reviewer has apparently misinterpreted our comment about open access publishing as a demand that “others should make their data available so that authors who use this method could use their method”. This is a simple misunderstanding. We are not discussing open data, but open access publishing. Discussing the benefits of open access publishing for meta-scientific research should not be a contentious or policital topic in a meta-scientific journal with a hybrid open access policy like Adolescent Research Review.


We have revised this section to avoid giving offense, and to strengthen our argument. The revision now reads:

> Another limitation is that our analysis was limited to keywords and abstracts. The primary obstacles to the analysis of full-text publications were limited access to articles behind paywalls, and the absence of a standardized Application Programming Interface (API) for the automatic retrieval of articles across publishing outlets.  Comprehensive access to scientific publications is essential to all meta-scientific research, including classic systematic reviews and meta-analyses (e.g., to avoid bias in summary effect sizes). For text mining approaches in particular, the availability of large data sets is crucial.  One example of a data set suitable for even more complex text mining analyses is a corpus of 400k+ full-text papers on COVID-19 that was made publicly available (Wang et al. 2020). Compiling such full-text data sets currently requires substantial manpower and financial resources, thus placing them out of reach for many researchers.   An increased adoption of open access publishing, and the development of a unified API for article retrieval across publishing outlets, would facilitate curating full-text data sets. These changes would enable more widespread adoption and more informative application of meta-scientific (text mining) analyses.

**Reviewer 1, comment 20:** in the end, it ends up looking like the authors are “hiding the ball”. The real issue is what is actually being added to theory? What are the important findings here? What are the practical findings that emerge from this process that are worth making this type of analysis. I just end up not convinced here.

**Response:** We regret that our writing raises these concerns about a lack of transparency, because we have made thorough efforts to ensure that this adheres to best practices for open science. For instance, we have published our data, code, and complete history of this project since its inception in an online repository. To make this abundantly clear, the revised manuscript now reads:

> we have made all code, data, and the historical record of this project publicly available in an online research repository at [masked]. We used the Workflow for Open Reproducible Code in Science (WORCS, **???**) to make the analyses strictly reproducible. We encourage reuse of the analysis code, and secondary analysis of the data.

Furthermore, the Reviewer understandably asks “what is actually being added to theory?”. The answer is that we are *not* adding to theory in the present paper, but we are *laying the groundwork* for theory construction. Because we did not seek to develop new theory, we had not gone into detail on theory construction methodology in the previous iteration of the manuscript.

We now see that this was a mistake, and that this literature provides necessary context for understanding the purpose of the study. We have thus attempted to address this comment by clarifying the relationship between inductive identification of relevant phenomena and formal theory construction:

> This has led to recent calls for the consolidation of empirical insights into overarching theory (e.g., Buss, Cole, and Zhou 2019). Formal methods for theory construction state that the first step in this endeavor is to identify the relevant *empirical phenomena* (**???**; **???**), defined as stable and general features of the world (**???**). We propose that systematic reviews are a suitable method for taking this first step, based on the assumption that phenomena considered to be stable and general will be more frequently represented in the published literature.

> The first step in theory construction methodology (TCM) is to identify relevant phenomena (**???**). We have argued that text mining systematic reviews are a relatively comprehensive and objective method for identifying such phenomena, because they can evaluate much larger bodies of literature than sentient readers, and are not biased toward pre-existing ideas.

The Reviewer further asks “What are the important findings here?”. To address this comment, we have added more extensive interpretations of the emerging themes in the Discussion and in the Abstract. For the sake of brevity, we just copy this excerpt from the Abstract to illustrate:

> Results reflected themes commonly featured in theory and narrative reviews, such as socialization and neurocognitive development, but also identified undertheorized themes, such as developmental disorders, physical health, external stressors, structural disadvantage, substance use, identity and moral development, and sexual development.

**Reviewer 1, comment 21:** The conclusion note that the method charts “unknown territories”. Sure that is not the case because the study relies on the published studies.

**Response:** The point is well-taken. We are not charting “unknown territories” at the level of primary research, but we are charting territories underrepresented at the meta-scientific level (i.e., topics rarely addressed in narrative reviews and theory). We have attempted to address this comment by rewriting the section as follows:

> Importantly, our results also draw attention to several relevant phenomena that, although well-represented in the empirical literature, are under-emphasized in narrative reviews and theory - such as developmental disorders, physical health, external stressors, structural disadvantage, substance use, identity and moral development, and sexual development.

**Reviewer 1, comment 22:** I really get a sense of over-selling. The authors should, again, focus on what is actually found that is useful about the topic. There is an example made of emerging themes and fringes, with the example given the little focus on fathers. That may be true, but there was not really a study needed to show that point, as it is well known that the field focuses on primary caretakers (mothers/women). The conclusion ends with noting that others have noted a need for an overarching theory; but that is what is the apparent contribution being made here; the author does not really get to that and address it; there is no sense of a contribution being made to theory.

**Response:** We agree that it is important to clearly communicate what is useful about the present study without over-selling. To address this point, we have carefully rewritten all claims about novel contributions.

We are not quite sure what to make of the comment that “there was not really a study needed to show that [there is little focus on fathers], as it is well known that the field focuses on primary caretakers (mothers/women)”. The Reviewer appears to suggest that we conducted this study in order to show that there is little focus on fathers - but this is not the case. We have emphasized throughout the manuscript that our study was inductive (data-driven), with no a-priori expectation about the phenomena we might find. We have simply reported the relevant phenomena that emerged from our analysis, and fathers are one of those phenomena. It seems relevant to discuss this particular finding in the context of prior work that has *argued* that fathers are underrepresented, but based on narrative reviews, not empirical analysis of the published literature.

We further think that the example of fatherhood is particularly instructive, especially because, as the Reviewer points out, “it is well known that the field focuses on primary caretakers (mothers/women)”. The fact that our analysis picks out a specific theme that is known by many researchers to be just outside the mainstream illustrates the method’s ability to detect fringe themes in general, and serves as an example to illustrate to readers how this analysis may also pick out other themes about which there is less consensus that they are underrepresented.

Finally, the Reviewer points out that “The conclusion ends with noting that others have noted a need for an overarching theory; but that is what is the apparent contribution being made here; the author does not really get to that and address it; there is no sense of a contribution being made to theory.” We have attempted to address this comment by clarifying the intended contribution of the present study (see response to comment R1C4). This specific section has been rewritten as follows:

> Finally, several authors have commented on the lack of an overarching theory tailored specifically to adolescent emotion regulation. The first step in theory construction methodology (TCM) is to identify relevant phenomena (**???**). We have argued that text mining systematic reviews are a relatively comprehensive and objective method for identifying such phenomena, because they can evaluate much larger bodies of literature than sentient readers, and are not biased toward pre-existing ideas. The next step in formal theory construction methodology would be to describe and quantify the nature of the relationship of each phenomenon in relation to adolescent emotion regulation. The phenomena identified in the present study might thus serve as a starting point for future theory development, and sensitize empirical researchers to potential confounders in research on adolescents’ emotion regulation.

Aldao, Amelia, Susan Nolen-Hoeksema, and Susanne Schweizer. 2010. “Emotion-Regulation Strategies Across Psychopathology: A Meta-Analytic Review.” *Clinical Psychology Review* 30 (2): 217–37.

Bariola, Emily, Eleonora Gullone, and Elizabeth K. Hughes. 2011. “Child and Adolescent Emotion Regulation: The Role of Parental Emotion Regulation and Expression.” *Clinical Child and Family Psychology Review* 14 (2): 198. https://doi.org/10.1007/s10567-011-0092-5.

Buss, Kristin A., Pamela M. Cole, and Anna M. Zhou. 2019. “Theories of Emotional Development: Where Have We Been and Where Are We Now?” In *Handbook of Emotional Development*, edited by Vanessa LoBue, Koraly Pérez-Edgar, and Kristin A. Buss, 7–25. Cham: Springer International Publishing. https://doi.org/10.1007/978-3-030-17332-6\_2.

Coe-Odess, Sarah J., Rachel K. Narr, and Joseph P. Allen. 2019. “Emergent Emotions in Adolescence.” In *Handbook of Emotional Development*, edited by Vanessa LoBue, Koraly Pérez-Edgar, and Kristin A. Buss, 595–625. Cham: Springer International Publishing. https://doi.org/10.1007/978-3-030-17332-6\_23.

Crone, Eveline A., and Ronald E. Dahl. 2012. “Understanding Adolescence as a Period of Social–Affective Engagement and Goal Flexibility.” *Nature Reviews Neuroscience* 13 (9): 636–50. https://doi.org/10.1038/nrn3313.

Lee, Francis S., Hakon Heimer, Jay N. Giedd, Edward S. Lein, Nenad Šestan, Daniel R. Weinberger, and B. J. Casey. 2014. “Adolescent Mental Health—Opportunity and Obligation.” *Science* 346 (6209): 547–49. https://doi.org/10.1126/science.1260497.

Littell, Julia H. 2008. “Evidence-Based or Biased? The Quality of Published Reviews of Evidence-Based Practices.” *Children and Youth Services Review* 30 (11): 1299–1317. https://doi.org/10.1016/j.childyouth.2008.04.001.

Sawyer, Susan M., Peter S. Azzopardi, Dakshitha Wickremarathne, and George C. Patton. 2018. “The Age of Adolescence.” *The Lancet. Child & Adolescent Health* 2 (3): 223–28. https://doi.org/10.1016/S2352-4642(18)30022-1.

Schäfer, Johanna Özlem, Eva Naumann, Emily Alexandra Holmes, Brunna Tuschen-Caffier, and Andrea Christiane Samson. 2017. “Emotion Regulation Strategies in Depressive and Anxiety Symptoms in Youth: A Meta-Analytic Review.” *Journal of Youth and Adolescence* 46 (2): 261–76. https://doi.org/10.1007/s10964-016-0585-0.

Silk, Jennifer S., Laurence Steinberg, and Amanda Sheffield Morris. 2003. “Adolescents’ Emotion Regulation in Daily Life: Links to Depressive Symptoms and Problem Behavior.” *Child Development* 74 (6): 1869–80. http://onlinelibrary.wiley.com/doi/10.1046/j.1467-8624.2003.00643.x/full.

Steinberg, Laurence. 2014. *Age of Opportunity: Lessons from the New Science of Adolescence*. Houghton Mifflin Harcourt. http://books.google.com?id=3cubAwAAQBAJ.

Stifter, Cynthia, and Mairin Augustine. 2019. “Emotion Regulation.” In *Handbook of Emotional Development*, edited by Vanessa LoBue, Koraly Pérez-Edgar, and Kristin A. Buss, 405–30. Cham: Springer International Publishing. https://doi.org/10.1007/978-3-030-17332-6\_16.

Wang, Lucy Lu, Kyle Lo, Yoganand Chandrasekhar, Russell Reas, Jiangjiang Yang, Darrin Eide, Kathryn Funk, et al. 2020. “CORD-19: The Covid-19 Open Research Dataset.” *ArXiv*, April. https://www.ncbi.nlm.nih.gov/pmc/articles/PMC7251955/.

Zimmermann, Peter, and Alexandra Iwanski. 2014. “Emotion Regulation from Early Adolescence to Emerging Adulthood and Middle Adulthood: Age Differences, Gender Differences, and Emotion-Specific Developmental Variations.” *International Journal of Behavioral Development* 38 (2): 182–94.
